# Ion Channels and Transporters in Disorders of Glucose Homeostasis: From Molecular Mechanisms to Precision Medicine

**DOI:** 10.3390/cells15212030

**Published:** 2026-11-06

**Authors:** Hajar Dauleh, Khalid Hussain

**Affiliations:** Division of Endocrinology, Department of Pediatric Medicine, Sidra Medicine, Doha P.O. Box 26999, Qatar; hdauleh1@sidra.org

**Keywords:** glucose homeostasis, ion channels, solute carriers, pancreatic islets, monogenic diabetes, hyperinsulinism, precision medicine

## Abstract

Glucose homeostasis requires coordinated nutrient sensing, membrane excitability, hormone secretion, tissue glucose flux, and mitochondrial metabolism. Ion channels and transporters connect these processes, but mechanistic importance does not necessarily establish human disease causality or clinical actionability. This narrative review integrates human islet electrophysiology with glucose, metal-ion, and mitochondrial transport, distinguishing established causal human disease, replicated genetic susceptibility, limited genetic observations, human functional evidence, and predominantly experimental mechanisms. ATP-sensitive potassium (K_ATP_) channel disorders provide the clearest bidirectional paradigm: activating *KCNJ11* or *ABCC8* variants can cause neonatal diabetes amenable to sulfonylureas, whereas inactivating variants cause congenital hyperinsulinism. Tissue-selective transporter syndromes, background potassium conductance, intercellular coupling, and organellar calcium handling illustrate additional mechanisms with different levels of human validation. Acquired remodeling in type 2 diabetes may be pathogenic, compensatory, or secondary to metabolic stress. We separate established management from investigational human replacement and preclinical targeting and critically assess functional assays, stem-cell models, transcriptomics, and genome engineering. Priorities are to resolve genotype–phenotype heterogeneity, establish causal direction and disease-stage dependence, and develop tissue-selective interventions with demonstrable patient benefit.

## 1. Introduction

Glucose homeostasis emerges from coordinated nutrient sensing, endocrine secretion, substrate transport, and inter-organ communication. Pancreatic β-cells increase insulin secretion as glucose rises, α-cells adapt glucagon output to nutrient availability and hypoglycaemia, and the intestine, kidney, liver, muscle, adipose tissue, and brain regulate glucose entry, disposal, storage, production, and behavioural responses [1,2,3,4]. Ion channels and transporters couple these processes by converting chemical gradients and metabolic signals into electrical and biochemical responses [1,2].

The β-cell pathway illustrates this integration. Glucose entry and metabolism raise the ATP/ADP ratio, close ATP-sensitive potassium (K_ATP_) channels, depolarize the plasma membrane, recruit voltage-gated calcium channels, and initiate calcium-dependent insulin-granule exocytosis [1,2]. Repolarizing potassium currents, sodium currents, intracellular calcium pumps and exchangers, mitochondrial transporters, and gap-junctional coupling determine the amplitude, timing, and synchrony of secretion [1,2,5]. This canonical model remains useful, but transporter expression, ion-channel complement, and electrical behaviour differ materially between human and rodent islets [1,3]. Mechanistic and therapeutic claims should therefore be anchored in human evidence whenever possible.

Human genetics has converted this physiology into clinical causality. Opposing functional changes in the K_ATP_-channel genes *KCNJ11* and *ABCC8* produce neonatal diabetes or congenital hyperinsulinism, and molecular diagnosis can determine whether sulfonylurea or diazoxide therapy is appropriate [6,7,8,9,10,11,12,13]. Disorders of *SLC2A1*, *SLC2A2*, *SLC5A1*, and *SLC5A2* demonstrate that the phenotype of transporter deficiency reflects tissue distribution rather than a uniform disturbance of blood glucose [14,15,16,17,18,19,20]. Conversely, common-variant associations at *KCNQ1* and *SLC30A8* identify biologically important pathways but do not, by themselves, establish a monogenic channelopathy or transportopathy [21,22,23,24].

This narrative review asks which channel and transporter mechanisms are established in humans, which remain model-dependent, and which can change clinical management. Our earlier review [25] surveyed inherited channelopathies and transportopathies associated with glucose disorders. The present article retains that disease foundation but changes the organizing question from a catalogue of affected genes to the relationship between physiological coupling, human evidence, and therapeutic utility. It integrates human islet electrophysiology, organellar transport, acquired remodeling, and peripheral insulin resistance, using separate classifications for human disease evidence, experimental validation, and intervention maturity. The intended conceptual advance is to identify where these evidence streams converge—and where a plausible mechanism still does not justify diagnostic attribution or treatment. It is a critical narrative synthesis, not an independently validated clinical scoring system.

### Literature Scope and Evidence Assessment

This narrative review was updated by a structured search of PubMed (NCBI, including MEDLINE records) on 23 October 2026. Eight Boolean search blocks covered K_ATP_-channel disease; other excitable channels; gap junctions and metabolic amplification; glucose transport; metal-ion and other disease-associated carriers; organellar transport; acquired human-islet dysfunction; and precision diagnosis or treatment. Gene symbols and pathway terms were combined with glucose, insulin, islet, or disease terms, primarily in title/abstract fields; the glucose-transport block also used the Humans MeSH term. Publications dated through 23 October 2026 were eligible, with no substantive lower date limit and no language filter. Animal and cellular studies were retained for mechanistic comparison. Embase, Scopus and Web of Science were not searched. Exact queries, execution dates, query translations, retrieval parameters and ordered block-specific PMID lists are provided in Supplementary Search Methods S1; original JSON responses and XML exports accompany it in Supplementary Search Audit S2.

Each Entrez ESearch request specified sort=relevance and retmax=60, with retstart omitted; all eight responses recorded retstart=0 and retmax=60. The 60 returned identifiers per block were retained in their original order before PMID-based deduplication. The 480 block-specific records represented 467 unique identifiers: 465 journal records and two book/database entries. AI-assisted title assessment prioritised 67 journal records, 63 with indexed abstracts, for closer assessment. Accessible full text was consulted selectively for key mechanistic claims, including Cx36 rescue, calcium-channel organisation and disputed *UCP2*–hyperinsulinism causality. Priorities were primary human genetic, human-islet and interventional evidence, variant-function studies, contradictory findings and clinical guidance; reviews supplied context, and relevant foundational references were retained. H.D. and K.H. verified the AI-assisted screening, key source interpretations and final H1–H5 assignments. This was not independent duplicate screening; no formal risk-of-bias assessment or meta-analysis was performed. Single-database coverage and relevance-ranked caps leave potentially relevant records unretrieved, including recent publications within the date range; the update is not comprehensive through the search date.

We use five descriptive human-evidence categories. H1 denotes an established causal human gene–disease relationship, supported by reproducible genotype–phenotype observations in independent families or cohorts, inheritance-compatible segregation or de novo occurrence, rarity consistent with the disorder, and a coherent mechanism; functional evidence strengthens attribution but need not come from human β-cells. H2 denotes replicated complex-trait association or an informative human allelic series without a conventional Mendelian dysglycaemia syndrome; linkage disequilibrium can leave the effector gene or direction unresolved. H3 denotes limited human genetic evidence, such as one or a few pedigrees with functional support but insufficient independent replication of the glucose phenotype. H4 denotes human physiological or functional evidence without an established causal genetic glucose disorder. H5 denotes predominantly animal, cell-line, or heterologous experimental evidence for the glucose mechanism. These categories distinguish kinds of evidence rather than forming a numerical score. They are an editorial synthesis, not formal ClinGen gene curation, ACMG variant classification, or GRADE assessment [26].

The assignment is phenotype-specific: an established systemic syndrome does not automatically establish a primary β-cell disorder. Tables distinguish systemic syndromes, glucose phenotypes, and measured human cellular functions and identify the functional model. Intervention maturity is assessed separately as established management, investigational in humans, or preclinical targeting; human ex vivo rescue does not establish patient benefit.

## 2. Integrated Electrical-Metabolic Coupling in the Pancreatic Islet

Stimulus–secretion coupling combines an electrical triggering pathway with metabolic amplification [1,2,5]. In the triggering pathway, glucose metabolism lowers K_ATP_ conductance sufficiently for inward currents to depolarize the membrane and recruit voltage-gated Ca^2+^ entry. Amplification increases exocytotic efficacy at a comparable cytosolic Ca^2+^ signal through metabolic coupling factors and granule competence; it is therefore not synonymous with further depolarization or higher global Ca^2+^. Experimentally, imposing comparable depolarization and Ca^2+^ while varying glucose helps separate these processes, although imperfect control of local Ca^2+^ microdomains limits interpretation. Paracrine and cAMP signals modulate both arms, so a secretion change cannot be assigned to one channel solely from insulin measurements.

Interpretation also requires attention to species. Mouse β-cells depend strongly on GLUT2 and differ from human β-cells in their balance of sodium and calcium currents; human β-cells use GLUT1 and GLUT3 more prominently and display substantial cell-to-cell variability [1,3,27]. Findings from knockout mice or immortalized cell lines are mechanistically valuable, but therapeutic inference should be tempered until replicated in primary human islets, genetically defined patients, or physiologically relevant stem-cell-derived models.

### 2.1. Conductance Balance and Dynamic Feedback

Membrane voltage reflects the sum of ionic currents, rather than K_ATP_ opening alone. The outward K^+^ current depends on both conductance and the difference between membrane potential and the K^+^ equilibrium potential. At low glucose, a large K_ATP_ conductance holds voltage relatively close to the K^+^ equilibrium potential and reduces input resistance. As K_ATP_ conductance falls, input resistance increases: a small inward current then produces a larger depolarization. Background K^+^ conductances and depolarizing currents set whether the threshold is reached. Remaining K_ATP_ conductance, extracellular K^+^, cell size, and electrical coupling therefore modify the effect of the same molecular perturbation [1,2,5].

During firing, Na^+^ and Ca^2+^ currents support depolarization, whereas voltage-gated and Ca^2+^-activated K^+^ currents limit action-potential duration and Ca^2+^ loading. Ca^2+^ entry also stimulates mitochondrial metabolism, which can sustain ATP production and further suppress K_ATP_ activity. ATP-consuming ion pumps and secretion oppose this positive feedback. Mitochondrial failure can leave K_ATP_ channels insufficiently inhibited despite elevated glucose; chronic Ca^2+^ overload can instead damage oxidative metabolism. Acute physiological feedback, persistent metabolic stress, and a constitutive genetic gating defect thus require different therapeutic interpretations [1,5,28,29]. Table 1 summarises these phases without implying a universal waveform across human donors.

### 2.2. Gap Junctions and Islet Network Coordination

Connexin 36, encoded by *GJD2*, forms gap junctions between β-cells. Primary human tissue studies demonstrate Cx36-containing junctions and intercellular coupling [30]. Electrical coupling distributes current between heterogeneous cells, so an excitable cell can recruit neighboring cells while less excitable neighbors can restrain inappropriate firing. Synchronized Ca^2+^ activity and pulsatile secretion are consequently properties of the coupled network, not simply the sum of isolated-cell channel currents [1,5]. Detailed loss-of-function experiments linking coupling to oscillatory organization are particularly strong in rodents; the degree and architecture of coupling in human islets require direct assessment. Correlations between *GJD2* and insulin expression do not establish a Mendelian diabetes mechanism or a clinically validated Cx36 therapy [30]. Islet dissociation removes this network context and can change the interpretation of single-cell patch-clamp and calcium-imaging results.

More recent experiments extend this beyond structural association: restoring Cx36 coupling improved coordinated Ca^2+^ dynamics and secretion in experimental preparations that included islets from donors with type 2 diabetes [31]. The responses varied across donors, and the interventions remain ex vivo tools rather than validated clinical Cx36 treatments. This provides a human functional rescue example without establishing that impaired coupling initiates diabetes. Figure 1 integrates these islet mechanisms with transport pathways across tissues. Table 2 summarizes the principal channels and transporters and the human evidence for their roles.

## 3. ATP-Sensitive Potassium Channels: The Clinically Actionable Paradigm

### 3.1. Structure and Glucose-Stimulated Insulin Secretion

The pancreatic K_ATP_ channel contains four Kir6.2 pore subunits, encoded by *KCNJ11*, and four SUR1 regulatory subunits, encoded by *ABCC8* [68,69]. Nucleotide inhibition at Kir6.2 is Mg^2+^-independent, whereas Mg-nucleotide interaction with the nucleotide-binding domains of SUR1 favors opening. MgADP stimulates through SUR1; MgATP can also support stimulatory nucleotide cycling, although net ATP action is normally inhibitory. Channel activity therefore integrates inhibitory ligand binding, SUR1-mediated activation, intrinsic gating, and surface channel number. Experiments that manipulate ATP must specify Mg^2+^ and nucleotide conditions to distinguish these effects [70].

At low glucose, substantial K_ATP_ conductance favors a negative membrane potential and low input resistance; glucose metabolism shifts the nucleotide balance towards channel inhibition, allowing depolarizing currents to recruit Ca^2+^ influx [1,2]. The ATP/ADP ratio is a useful physiological shorthand, not a complete description of the free nucleotides and Mg-bound species at the channel. A variant can reduce apparent ATP sensitivity by changing ATP binding, allosteric gating, Mg-nucleotide activation, or trafficking. Thus, not every activating variant directly disrupts the ATP-binding site [68,69,70].

### 3.2. Activating Variants and Neonatal Diabetes

Gain-of-function variants in *KCNJ11* and *ABCC8* are among the most common genetic causes of neonatal diabetes mellitus (NDM); their relative frequency depends on whether transient or permanent disease is considered and on population structure [6,9,13]. Increased channel activity can reflect impaired inhibitory nucleotide binding, enhanced open-state stability, altered SUR1 regulation, or combinations of these mechanisms. Persistent outward K^+^ current prevents glucose-dependent depolarisation, limiting Ca^2+^ entry and insulin secretion despite metabolically available glucose [6,7,8,9,10,70].

The clinical phenotype encompasses a broad spectrum of disease severity, ranging from isolated diabetes to complex neurodevelopmental syndromes. Patients may present with transient neonatal diabetes (TNDM), in which hyperglycemia resolves during infancy but may recur later in life, or permanent neonatal diabetes mellitus (PNDM), which requires lifelong treatment [6,7,8,9,10]. At the severe end of the spectrum are intermediate developmental delay, epilepsy, and neonatal diabetes (intermediate DEND) syndrome and classic DEND syndrome, characterized by PNDM accompanied by developmental delay, epilepsy, hypotonia, and other neurological abnormalities [6,7,8,9,10]. The marked phenotypic variability reflects the degree of K_ATP_ channel overactivity conferred by the underlying variant [6,7,8,9,10]. Variants causing greater resistance to ATP-mediated inhibition produce more severe β-cell dysfunction and are associated with neurological involvement due to persistent activation of K_ATP_ channels in the brain, where these channels play important roles in regulating neuronal excitability and neurotransmission [6,7,8,9,10]. Activating K_ATP_-channel variants may also present beyond infancy as *ABCC8*- or *KCNJ11*-related maturity-onset diabetes of the young, and some dominant inactivating *ABCC8* variants cause hyperinsulinemic hypoglycaemia in infancy followed by diabetes in later life [13,25].

Later-onset *ABCC8*-associated diabetes should not automatically be interpreted as channel gain of function. A recent recombinant-channel analysis of variants reported with maturity-onset diabetes found predominantly loss-of-function effects [71]. This reinforces the need to integrate variant function and clinical history before extrapolating the sulfonylurea paradigm of activating neonatal diabetes variants to every *ABCC8*-associated adult phenotype.

### 3.3. Inactivating Variants and Congenital Hyperinsulinism

In contrast to gain-of-function variants that cause neonatal diabetes, loss-of-function variants in *ABCC8* or *KCNJ11* result in inactivation of the β-cell K_ATP_ channel, preventing normal channel opening during hypoglycaemia [11,12,13]. The consequent reduction in potassium efflux leads to persistent β-cell membrane depolarization, constitutive activation of voltage-dependent calcium channels, sustained intracellular calcium influx, and inappropriate insulin secretion despite low plasma glucose concentrations [11,12]. Biallelic recessive variants typically cause severe diffuse, diazoxide-unresponsive disease, whereas dominant inactivating variants can be diazoxide-responsive or unresponsive, depending on their functional effects; dominant inheritance alone does not predict mild disease. Focal disease arises when a paternally inherited *ABCC8* or *KCNJ11* variant is unmasked by somatic loss of the maternal 11p15 region within a discrete area of the pancreas [11,12,25].

K_ATP_ channel-related CHI is the most common and often the most severe genetic form of hyperinsulinemic hypoglycaemia [11,12]. Clinically, affected infants present with recurrent or persistent hypoglycaemia, inappropriately elevated insulin secretion, suppressed ketogenesis and lipolysis, and an increased risk of seizures and permanent neurodevelopmental injury if diagnosis and treatment are delayed [11,12].

The clinical phenotype ranges from medically responsive diffuse disease to severe diazoxide-unresponsive forms that may require surgical intervention [11,12]. Establishing the underlying molecular diagnosis is fundamental to the management of CHI, as the causative genotype influences disease severity, responsiveness to diazoxide, the likelihood of focal versus diffuse pancreatic disease, the need for 18F-fluoro-L-DOPA positron emission tomography imaging, and decisions regarding partial or near-total pancreatectomy [11,12]. Consequently, genetic testing has become an integral component of precision diagnosis and individualized treatment in CHI.

### 3.4. Precision Pharmacology

Activating *KCNJ11* or *ABCC8* variants often retain pharmacological responsiveness to sulfonylureas, which bind SUR1 and close the channel independently of ATP, enabling membrane depolarization and endogenous insulin release [6,7,8,9,10]. Transfer should follow a genotype-informed protocol with close glucose monitoring because dose requirements are often higher than in type 2 diabetes and response varies with functional severity [72]. Long-term cohort data support durable glycemic control in most successfully transferred patients; neurological improvement is less consistent and appears more likely when treatment begins early [7,8,10].

Diazoxide has the opposite pharmacological objective: it stabilizes the open K_ATP_ channel and suppresses insulin release. It is effective only when sufficient channel function remains and is therefore less likely to work in severe biallelic loss-of-function disease [11,12]. Genotype, 18F-DOPA imaging where indicated, and clinical responsiveness together distinguish focal from diffuse congenital hyperinsulinism and guide medical versus surgical management [11,12]. Table 3 compares the opposing consequences of gain- and loss-of-function variants.

## 4. Voltage-Gated Calcium and Potassium Channels

### 4.1. Calcium Entry, Exocytosis, and Oscillations

Primary human β-cell electrophysiology identifies L-type Ca_V_1.2/Ca_V_1.3, P/Q-type Ca_V_2.1, and T-type Ca_V_3.x contributions, with donor- and protocol-dependent current proportions [1,5,35]. Low-voltage-activated T-type current can influence recruitment of firing, while L-type and P/Q-type currents contribute during depolarization. In the human preparations examined by Braun and colleagues, P/Q-type influx was especially effective at coupling to exocytosis [1]. This does not establish universal dominance across all human β-cells: Ca^2+^ entry near granule-release sites, charge carried during an action potential, and the global cytosolic Ca^2+^ signal are different measurements. Ca_V_1.2 and Ca_V_1.3 cannot be treated as interchangeable based on L-type pharmacology alone, and Ca_V_3.2 findings should be interpreted according to the human-islet and model-system experiments actually performed [35].

Complementary high-resolution studies identify L-type Ca^2+^ channels clustered near release-ready granules in human β-cells and INS1 cells; disrupted organization in diabetic donor β-cells supports the importance of release-site architecture [73]. This prevents a universal assignment of the exocytotic trigger to P/Q-type channels. The relevant questions are which channels couple to a specific granule pool, under which preparation and stimulus, and whether their spatial association is preserved in diabetes.

The amplitude, spatial distribution, and timing of calcium signals shape the biphasic and pulsatile pattern of insulin secretion [1,5]. Rapid local calcium entry releases the readily releasable granule pool, whereas sustained and oscillatory signals support reserve-granule recruitment. Gap-junctional coupling coordinates heterogeneous β-cells across the islet, synchronizing electrical and secretory oscillations [1,5].

Disrupted calcium dynamics are a feature of β-cell dysfunction in type 2 diabetes and during chronic glucotoxicity or lipotoxic stress [55,74]. Abnormal influx and defective organellar calcium handling reduce pulsatile secretion and promote endoplasmic-reticulum stress, mitochondrial dysfunction, and apoptosis [55,74]. Therapeutic strategies must therefore restore physiological calcium dynamics without causing chronic calcium loading.

### 4.2. Human Calcium-Channel Disease and Evidence Limits

*CACNA1D* encodes Ca_V_1.3, an L-type calcium channel expressed in β-cells, the adrenal zona glomerulosa, the sinoatrial node, and the nervous system [33]. Activating variants cause a multisystem phenotype dominated by primary aldosteronism and neurodevelopmental disease; hyperinsulinemic hypoglycaemia has been reported in selected patients [33,34]. This provides human evidence that excessive Ca_V_1.3 activity can disturb endocrine secretion, but *CACNA1D* is not a common cause of isolated hyperinsulinism or diabetes. Similarly, gain-of-function *CACNA1C* variants cause Timothy syndrome, a multisystem disorder of Ca_V_1.2 dysfunction in which intermittent hypoglycaemia occurs alongside cardiac arrhythmia, syndactyly, and autism spectrum features [32]. Evidence for other VGCCs in dysglycaemia remains predominantly experimental.

### 4.3. Repolarizing Potassium Currents

Voltage-gated potassium (K_v_) channels shape action-potential duration, calcium entry, and insulin secretion by repolarizing the β-cell membrane after depolarization [38,39]. Their pore-forming α-subunits assemble as homo- or heterotetramers and are modulated by auxiliary subunits that affect trafficking and gating. Human β-cells express K_v_2.1, K_v_7.1, and other K_v_ subtypes, but their relative contributions differ across species and experimental models [1,38,39]. Premature repolarization limits calcium entry, whereas prolonged depolarization can initially augment secretion but ultimately increase calcium stress.

K_v_2.1 has separable conducting and organizational functions. Its K^+^ current can shorten depolarization, whereas membrane clusters can organize granule recruitment and exocytosis independently of simply carrying more repolarizing current. Fu and colleagues demonstrated clustering-dependent effects and rescue of exocytotic dysfunction in human β-cells [38]; SUMOylation studies provide additional experimental evidence for regulation of gating [39]. Acute pore blockade, deletion of the protein, and disruption of its clustering are therefore different interventions and need not have the same secretory outcome. A therapy that preserves the exocytotic scaffold while modifying current remains a hypothesis, constrained by cardiac and neuronal expression.

Human genetics supports a role for repolarizing potassium channels in glucose homeostasis. Loss-of-function variants in *KCNH2*, which encodes the K_v_11.1 (hERG) channel, cause long-QT syndrome type 2 and are associated with exaggerated postprandial insulin and incretin secretion and reactive hypoglycaemia [37], and variants in *KCNH6* (K_v_11.2) have been reported in a family with hyperinsulinemic hypoglycaemia and later-onset diabetes [25]. A gain-of-function variant in *KCNK16*, encoding the two-pore-domain potassium channel TALK-1, has been associated with maturity-onset diabetes of the young, identifying a background potassium conductance as a candidate monogenic diabetes gene [40].

#### Background K^+^ Conductance and TALK-1

Two-pore-domain K^+^ channels provide a background conductance that can influence threshold and interburst voltage before the strong depolarization needed to recruit many voltage-gated K^+^ currents. They are not simply delayed repolarizing channels. TALK-1 (K2P16.1), encoded by *KCNK16*, can oppose glucose-dependent depolarization and alter Ca^2+^ entry and secretion. A segregating gain-of-function variant has functional support, including reduced secretion in transduced human islets and membrane-potential and Ca^2+^ experiments in mouse islets [40]. Human genetic replication remains limited, and overexpression does not establish the quantitative contribution of endogenous TALK-1 in every human β-cell. It is nevertheless a mechanistically distinct background-current target, not a sulfonylurea-sensitive substitute for K_ATP_.

TALK-1 also illustrates communication between plasma-membrane excitability and organellar Ca^2+^: experiments, especially in mouse β-cells, implicate an ER-localized pool in counter-ion flux and ER Ca^2+^ handling [41]. Its effects on initiation of firing, bursting, and ER stores must therefore be separated experimentally. Neither disease association nor model rescue yet defines a validated TALK-1-directed treatment for patients.

### 4.4. KCNQ1 and Complex-Trait Susceptibility

*KCNQ1* is a reproducible type 2 diabetes susceptibility locus, particularly in East Asian ancestry groups [21,22,75]. The locus lies in an imprinted region at 11p15.5, and risk may reflect developmental, parent-of-origin, or regulatory effects involving neighboring transcripts as well as altered K_v_7.1 activity [22]. It is therefore more accurate to describe *KCNQ1* as a complex-trait locus with plausible β-cell mechanisms than as a monogenic diabetes channelopathy. Conversely, loss-of-function *KCNQ1* variants causing long-QT syndrome type 1 are associated with hyperinsulinemia and symptomatic postprandial hypoglycaemia, supporting a direct contribution of K_v_7.1 to human β-cell repolarization [36].

### 4.5. Calcium-Activated Potassium Channels

BK channels (KCa1.1; *KCNMA1*) have large single-channel conductance and are activated jointly by depolarization and local Ca^2+^. SK channels (KCa2.1–KCa2.3; *KCNN1*–*KCNN3*) have small conductance and largely voltage-independent Ca^2+^/calmodulin-mediated activation. KCa3.1 (*KCNN4*) has intermediate conductance and is also regulated through Ca^2+^/calmodulin rather than BK-like voltage sensing. BK can therefore provide rapid feedback during an action potential, whereas SK and KCa3.1 can influence after hyperpolarization, interspike timing, or bursting according to their localization and Ca^2+^ exposure [1,2,42]. These functional assignments are context-dependent, not exclusive signatures of each family.

BK participation in repolarization is supported directly by primary human β-cell recordings [1]. More detailed assignments of SK and KCa3.1 to burst organization rely substantially on mouse islets and cellular models [42]; expression alone does not prove an equivalent endogenous current in human β-cells. Pharmacological interpretation requires concentration and selectivity controls: iberiotoxin or paxilline sensitivity supports BK involvement, apamin-sensitive current supports susceptible SK isoforms, and KCa3.1 inhibition requires appropriate controls for compounds such as TRAM-34. None alone proves the relevant gene or excludes off-target actions. A convincing subtype assignment combines current kinetics and voltage/Ca^2+^ dependence with genetic loss and rescue, protein localization, and orthogonal pharmacology. Insulin release alone is insufficient to assign a particular KCa current or infer that chronic blockade is beneficial.

Pathogenic *KCNMA1* and *KCNN* variants primarily cause neurological channelopathies rather than a reproducible dysglycaemia syndrome [42,43]. These genes should therefore be considered mechanistic regulators and experimental targets, not established monogenic diabetes genes [42,43]. Tissue-selective modulation and confirmation in human islets are prerequisites for therapeutic development.

## 5. Voltage-Gated Sodium Channels and Counter-Regulation

Voltage-gated sodium (Na_V_) channels are not the principal metabolic sensors in β-cells, but they shape action-potential threshold and upstroke and thereby influence downstream calcium entry [1,44]. Their contribution is species- and cell-type-dependent, which explains why sodium currents may appear dispensable in some rodent preparations yet remain functionally relevant in human islets [1,44].

### 5.1. Sodium Currents in β-Cells

Primary human β-cell recordings demonstrate functional voltage-gated Na^+^ currents [1]. Isoform expression and availability depend on cell identity, membrane potential, and preparation; Na_V_1.3 (*SCN3A*), Na_V_1.6 (*SCN8A*), and Na_V_1.7 (*SCN9A*) should be regarded as representative candidates rather than uniformly established dominant human β-cell isoforms. The isoform-dissection and genetic evidence in reference [44] relies substantially on mouse islet cells and expression systems and must not be presented as a direct map of human β-cell current. Na^+^ current can accelerate the upstroke and recruit Ca^2+^ entry when channels recover from inactivation, but sustained depolarisation reduces availability; its contribution therefore changes with the resting and interspike potentials [1,44].

### 5.2. Alpha-Cell Excitability and Neural Counter-Regulation

Counter-regulation during falling glucose concentrations integrates central glucose-sensing circuits, autonomic output, adrenal catecholamine release, and pancreatic α-cell glucagon secretion [76]. Na_V_ channels support neuronal firing within these circuits and contribute to the detection and coordinated correction of hypoglycaemia [44].

Na_V_ channels also contribute to α-cell action potentials and calcium-dependent glucagon exocytosis [44,77]. Their effect depends on membrane potential because excessive depolarization can inactivate sodium and calcium currents, providing one mechanistic explanation for impaired glucagon secretion at high glucose [77]. In diabetes, altered α-cell excitability may contribute to both inappropriate glucagon secretion during hyperglycemia and defective responses to hypoglycaemia.

Network mechanisms also contribute to counter-regulation. Work in human type 1 diabetic donor islets and mouse models supports loss of a β-cell-to-δ-cell electrical restraint on somatostatin secretion, with consequent suppression of low-glucose glucagon release [78]. The cross-cell circuit complements intrinsic α-cell excitability and illustrates why correction of an isolated current need not restore whole-islet counter-regulation.

Islet sodium, potassium, and calcium currents should therefore be considered as an integrated network across endocrine cell types [44,76,77]. Inherited *SCN9A* and *SCN5A* disorders primarily cause sensory-neuronal and cardiac phenotypes, respectively; neither is an established cause of monogenic diabetes. By contrast, the chloride channel *CFTR* provides a clear example of a Mendelian channelopathy with frequent dysglycaemia: cystic fibrosis-related diabetes is the most common extrapulmonary comorbidity of cystic fibrosis, reflecting islet loss within the damaged exocrine pancreas together with possible intrinsic effects of *CFTR* dysfunction on β-cell function [45]. Direct Na_V_-channel therapy for dysglycaemia is currently constrained by neurological and cardiac off-target effects.

## 6. Glucose Transport Across Tissues

### 6.1. Facilitative Glucose Transporters

Because glucose cannot freely cross lipid bilayers, its movement between extracellular and intracellular compartments depends on specialized transport proteins [3,4]. Facilitative glucose transporters (GLUTs; SLC2 family) move substrates down concentration gradients, whereas sodium-glucose cotransporters (SGLTs; SLC5 family) use the sodium gradient to drive uptake. Tissue distribution, affinity, capacity, and subcellular localization determine whether a defect presents as neurological disease, altered glucose sensing, intestinal malabsorption, renal glucosuria, or insulin resistance [3,4,14,15,16,17,18,19,20,50].

Across the GLUT family, tissue distribution and regulated membrane localization are more clinically informative than a catalogue of shared structural features. Established human transporter disorders identify the affected organ; acquired trafficking defects require separate evidence of causality and treatment response [3,14,15,16,50].

GLUT1 (*SLC2A1*) supports basal glucose delivery in several tissues, including the blood–brain barrier and human β-cells [3,14,15,27]. Its broad expression does not imply that its inherited deficiency causes systemic diabetes; the dominant human phenotype reflects cerebral glucose deprivation.

Pathogenic variants in *SLC2A1* cause GLUT1 deficiency syndrome (GLUT1DS), a rare neurometabolic disorder resulting from impaired glucose transport across the blood–brain barrier [14,15]. The disorder is characterized by cerebral energy deficiency despite normal or only mildly reduced blood glucose concentrations, owing to inadequate glucose delivery to the central nervous system. Clinically, patients typically present with early-onset epilepsy, developmental delay, movement disorders, acquired microcephaly, and varying degrees of intellectual disability [14,15]. A hallmark biochemical feature is hypoglycorrhachia, with reduced cerebrospinal fluid glucose concentrations in the presence of normal or near-normal plasma glucose levels.

Although diabetes mellitus is not a recognized feature of GLUT1 deficiency syndrome, the disorder illustrates the fundamental importance of tissue-specific glucose transport in maintaining cellular energy homeostasis. Current management is centred on ketogenic diet therapy, which provides ketone bodies as an alternative cerebral energy substrate, together with avoidance of prolonged fasting to minimize metabolic stress [14,15,79]. Emerging therapeutic strategies, including approaches aimed at enhancing GLUT1 expression or function, may further improve neurological outcomes in the future.

### 6.2. GLUT2 and Fanconi-Bickel Syndrome

GLUT2, encoded by *SLC2A2*, is a low-affinity, high-capacity facilitative transporter expressed prominently in hepatocytes, renal tubular epithelium, and intestinal cells and at lower levels in human than rodent β-cells [3,16]. Its bidirectional transport supports hepatic glucose uptake and release, renal glucose handling, and intestinal transport. Rodent β-cells depend strongly on GLUT2 for glucose sensing, whereas human β-cells rely more heavily on GLUT1 and GLUT3 [1,3,27].

Loss-of-function variants in *SLC2A2* cause Fanconi–Bickel syndrome, a rare autosomal recessive disorder characterized by impaired glucose transport and glycogen metabolism [16]. The clinical phenotype includes hepatomegaly, renal Fanconi syndrome, fasting hypoglycaemia, postprandial hyperglycemia, excessive glycogen accumulation in the liver and kidneys, and growth failure [16]. These manifestations reflect the essential role of GLUT2 in hepatic glucose uptake and release, renal glucose handling, and the maintenance of systemic glucose homeostasis.

Human *SLC2A2*-related disease establishes a causal transport disorder with combined hepatic and renal effects, rather than proving that human β-cell sensing simply reproduces rodent GLUT2 dependence. Neonatal diabetes has been reported with biallelic *SLC2A2* variants [49], but diabetes is not the defining feature of all Fanconi–Bickel cases. Clinically actionable care remains nutritional and renal/metabolic management, with treatment of diabetes when present [16,49].

### 6.3. GLUT4 and Insulin-Responsive Glucose Disposal

GLUT4, encoded by *SLC2A4*, is the principal insulin-responsive transporter in skeletal muscle, cardiac muscle, and adipose tissue [50]. Insulin receptor–PI3K–AKT signaling mobilizes GLUT4 storage vesicles to the plasma membrane, markedly increasing postprandial glucose disposal. Exercise and contraction can also recruit GLUT4 through partly insulin-independent pathways. Defects in transporter abundance, vesicle trafficking, or upstream signaling therefore contribute directly to insulin resistance.

Impaired GLUT4 translocation in type 2 diabetes reflects interacting defects in insulin signaling, chronic inflammation, lipotoxicity, and mitochondrial function [50]. Because skeletal muscle is the principal site of insulin-stimulated glucose disposal, reduced GLUT4 availability promotes persistent hyperglycemia and compensatory hyperinsulinemia. This acquired pathophysiology differs from the Mendelian tissue-selective transporter disorders caused by *SLC2A1* or *SLC2A2* variants.

GLUT1 deficiency and Fanconi-Bickel syndrome illustrate tissue-selective transporter disease; GLUT4 dysfunction illustrates acquired insulin resistance [14,15,16,50]. These mechanisms should be distinguished from weaker or indirect metabolic associations involving other SLC2 members. GLUT9 is primarily a urate transporter and GLUT10 deficiency causes arterial tortuosity syndrome; neither currently supports a major direct role as a glucose-disorder gene [3].

### 6.4. Sodium-Glucose Cotransporters

Sodium-glucose cotransporters use the sodium electrochemical gradient generated by the Na^+^/K^+^-ATPase to move glucose against its concentration gradient. SGLT1 (*SLC5A1*) mediates intestinal glucose and galactose absorption and reclaims residual filtered glucose in the S3 proximal tubule, whereas SGLT2 (*SLC5A2*) reabsorbs most filtered glucose in the S1/S2 proximal tubule [4,17,18,19,20].

SGLT1 also participates in intestinal glucose sensing and incretin release, linking luminal nutrient uptake to postprandial endocrine responses [4,17,18]. Its tissue distribution and transport stoichiometry differ from SGLT2, explaining why inherited defects of the two transporters produce distinct phenotypes.

Biallelic *SLC5A1* variants cause glucose-galactose malabsorption, which presents in early infancy with severe osmotic diarrhea, dehydration, hypernatremia, and failure to thrive after glucose- or galactose-containing feeds; symptoms improve when these sugars are replaced by fructose [17,18].

Loss-of-function *SLC5A2* variants cause familial renal glucosuria, typically an isolated and benign glucosuria with normal blood glucose and preserved kidney function [19,20]. This human phenotype provided physiological proof of concept for therapeutic SGLT2 inhibition [19,20,51,52].

### 6.5. SGLT2 Inhibition as Physiology Translated into Therapy

SGLT2 inhibitors reduce renal glucose reabsorption and lower glycaemia through an insulin-independent mechanism. Outcome trials established cardiovascular and kidney protection that extends beyond modest reductions in HbA1c, body weight, and blood pressure, including in selected patients without diabetes [51,52].

Their kidney effects include restoration of tubule-glomerular feedback, reduction in intraglomerular pressure, and attenuation of albuminuria and progressive loss of kidney function [51,52]. Because glucosuria diminishes as plasma glucose approaches the normal range, the intrinsic risk of hypoglycaemia is low unless SGLT2 inhibitors are combined with insulin or insulin secretagogues. Improvements in β-cell function are likely indirect consequences of reduced glucotoxicity rather than direct islet channel modulation.

### 6.6. Other Solute Carriers Causing Monogenic Dysglycaemia

Several additional solute carriers cause Mendelian dysglycaemia through mechanisms other than glucose flux. Monocarboxylate transporter 1 (MCT1), encoded by *SLC16A1*, is normally silenced in β-cells; dominant promoter variants that prevent this silencing allow pyruvate and lactate generated during anaerobic exercise to enter β-cells and trigger inappropriate insulin secretion, causing exercise-induced hyperinsulinemic hypoglycaemia [80]. Biallelic *SLC19A2* variants impair high-affinity thiamine transport and cause thiamine-responsive megaloblastic anemia with diabetes mellitus and sensorineural deafness; pharmacological thiamine can improve glycaemia, although many patients eventually require insulin [25,81]. Biallelic *SLC29A3* variants, affecting the equilibrative nucleoside transporter ENT3, cause pigmented hypertrichosis with insulin-dependent diabetes (PHID) syndrome within the broader H-syndrome spectrum [82]. These disorders show that transporter defects can alter β-cell function through fuel selection and cofactor or nucleoside availability, not only through glucose transport.

## 7. Zinc, Magnesium, and Intracellular Calcium Transport

### 7.1. Zinc Transport and ZnT8

Metal-ion transport complements membrane excitability by regulating insulin biosynthesis, granule maturation, intracellular signaling, mitochondrial metabolism, and cell survival. Zinc, magnesium, and calcium therefore influence glucose homeostasis through compartment-specific transport rather than through interchangeable bulk changes in cellular ion content [48,54,56,59,60].

In β-cell secretory granules, zinc stabilizes insulin hexamers and is co-released with insulin, where it may contribute to paracrine islet signaling [54]. Cytosolic and organellar zinc are coordinated by the ZnT (SLC30) and ZIP (SLC39) families: ZnT proteins lower cytosolic zinc by export or sequestration, whereas ZIP proteins raise cytosolic zinc through influx or mobilization from intracellular stores [23,24,54,83].

ZnT8, encoded by *SLC30A8*, transports zinc into insulin granules and influences insulin crystallization, granule ultrastructure, and secretion [23,24,54]. Human genetic evidence is directionally complex: the common rs13266634 variant (p.Arg325Trp) is associated with type 2 diabetes susceptibility, with the Arg325 allele conferring increased risk [53], whereas rare protein-truncating variants and complete loss of function can protect against disease or improve glucose metabolism [23,24]. This allelic series supports ZnT8 as a causal susceptibility pathway but not as a conventional monogenic diabetes gene.

ZnT8 is also a major type 1 diabetes autoantigen. ZnT8 autoantibodies increase the sensitivity of islet autoantibody panels and can assist classification and risk assessment when interpreted with other autoantibodies and the clinical phenotype [83,84]. Therapeutic ZnT8 modulation will require dose and tissue calibration because zinc transport affects both granule biology and immune recognition.

Zinc accumulation has been linked to an ATF4–ARX stress and identity-loss pathway in stem-cell-derived islets, alongside human/mouse islet observations [85]. Perturbation and graft experiments support this experimental mechanism; whether it mediates protection from specific *SLC30A8* alleles remains unresolved.

### 7.2. Magnesium Transport

Magnesium supports ATP synthesis and utilization, insulin-receptor signaling, GLUT4 translocation, glycolysis, oxidative phosphorylation, and glucose-stimulated insulin secretion [48]. Deficiency can impair both β-cell function and peripheral insulin sensitivity, but these effects do not establish a primary genetic diabetes syndrome. *TRPM6* is expressed principally in intestinal epithelium and the renal distal convoluted tubule; biallelic loss-of-function variants cause hypomagnesaemia with secondary hypocalcemia, typically presenting in infancy with seizures, tetany, or arrhythmia [46,47]. Disturbed glucose handling may accompany severe deficiency, whereas direct evidence that *CNNM2* or *SLC41A1* variants cause human diabetes remains insufficient [46,47,48].

*TRPM6*-related disease establishes a role in intestinal/renal Mg^2+^ handling; direct β-cell roles of *CNNM2* and *SLC41A1* remain less secure. Mg^2+^ affects ATP-dependent metabolism, Mg-independent Kir6.2 inhibition and Mg-nucleotide-dependent SUR1 activation. Total serum or cellular Mg^2+^ does not quantify free cytosolic Mg^2+^ or local MgATP/MgADP at the channel [46,47,48,70]. Mg^2+^ replacement treats deficiency rather than a demonstrated transporter-selective β-cell defect.

### 7.3. Endoplasmic-Reticulum and Plasma-Membrane Calcium Handling

Cytosolic calcium is the final trigger for insulin-granule exocytosis, but the signal must be rapidly redistributed or removed after secretion. Sarco/endoplasmic reticulum Ca^2+^-ATPases (SERCAs), plasma-membrane Ca^2+^-ATPases (PMCAs), Na^+^/Ca^2+^ exchangers, and mitochondrial transport systems therefore shape the amplitude, duration, and compartmentalization of calcium responses [28,29,55,56,57,58,59,60,74]. Chronic calcium elevation promotes endoplasmic-reticulum stress, mitochondrial dysfunction, oxidative stress, and apoptosis.

SERCA2b (*ATP2A2*) and SERCA3 (*ATP2A3*) replenish ER Ca^2+^ stores. PDX1–SERCA2b experiments and mouse-islet measurements support a feedback loop in which ATP depletion impairs pumping, while ER Ca^2+^ loss compromises protein folding and secretion [55,56]. Whether reduced SERCA initiates human type 2 diabetes remains unresolved; proposed interventions should measure compartment-specific Ca^2+^ handling.

Plasma-membrane Ca^2+^-ATPases (PMCAs) extrude cytosolic Ca^2+^, alongside gradient-dependent Na^+^/Ca^2+^ exchange [57,58]. Partial PMCA-inactivation and clearance experiments in mice or mouse β-cells show acute secretory effects, with chronic benefit unresolved. Exocytotic microdomains, bulk cytosolic, ER luminal and mitochondrial matrix Ca^2+^ are distinct readouts and should be measured separately.

Mitochondria integrate cytosolic calcium with oxidative metabolism. Calcium enters through the MCU complex and is extruded principally by NCLX, shaping matrix calcium transients and the activity of calcium-sensitive dehydrogenases [28,29,59,60]. Insufficient uptake weakens metabolic amplification, whereas sustained accumulation promotes reactive oxygen species and loss of mitochondrial function. The therapeutic objective is therefore restoration of stimulus-coupled flux rather than indiscriminate enhancement or suppression of calcium entry.

## 8. Mitochondrial Transporters and Metabolic Coupling

Pancreatic β-cell mitochondria generate the ATP signal that initiates electrical activity and the metabolites that amplify exocytosis. This coupling depends on coordinated substrate transport, tricarboxylic-acid-cycle flux, redox shuttles, nucleotide exchange, calcium handling, and respiratory ATP production [59,60,61,62,63,64,65,66].

The mitochondrial pyruvate carrier (*MPC1*/*MPC2*) links glycolytic pyruvate to oxidation and anaplerosis, supporting glucose-stimulated insulin secretion in islet and cellular experiments [62,63]. Adenine nucleotide translocase (ANT) exchanges matrix ATP and cytosolic ADP, while phosphate import supports ATP synthesis. Parallel ATP, electrical, Ca^2+^ and secretory measurements are needed to distinguish effects on K_ATP_-dependent triggering from metabolic amplification [28,62,63,64,65].

*VDAC1* illustrates the importance of location. Work in diabetic human islets and models implicates pathological surface translocation and ATP loss; inhibition improves secretion ex vivo [61]. Selective inhibition of surface *VDAC1* may have a different therapeutic window from suppression of essential mitochondrial exchange.

The mitochondrial calcium uniporter (MCU) complex, including *MICU1*, *MICU2*, and EMRE (*SMDT1*), links cytosolic Ca^2+^ signals to matrix uptake [28,59,60]. Much of the detailed causal evidence comes from rodent β-cells and cellular perturbation. Physiological matrix Ca^2+^ can support oxidative metabolism and sustain the ATP signal that closes K_ATP_ channels, but prolonged loading can impair organelle function. Hence, mitochondrial uptake can feed back positively on secretion acutely and contribute to failure under chronic stress; a larger Ca^2+^ transient is not intrinsically beneficial.

The mitochondrial Na^+^/Ca^2+^ exchanger NCLX (*SLC8B1*) shapes recovery of matrix Ca^2+^ between stimuli; its perturbation alters Ca^2+^ kinetics and insulin secretion in β-cell models [29]. MCU uptake and NCLX extrusion must be assessed together to distinguish metabolic support from persistent matrix loading.

*UCP2* can reduce mitochondrial coupling efficiency and the ATP/ADP signal required for insulin secretion, yet moderate uncoupling may limit reactive oxygen species during chronic metabolic stress [66]. Its net effect is context dependent. Heterozygous *UCP2* variants have been reported in children with diazoxide-responsive congenital hyperinsulinism [86], but a causal role has not been consistently replicated. *SLC25A3* imports phosphate required for ATP synthesis, whereas *SLC25A1* exchanges citrate and related tricarboxylates between the matrix and cytosol [64]. These functions are established biochemically, but their quantitative contribution to human β-cell secretion is less secure than that of the mitochondrial pyruvate carrier; claims of dysglycaemia should distinguish multisystem mitochondrial disease from isolated β-cell phenotypes.

The proposed *UCP2*–hyperinsulinism relationship has also been challenged directly. Analysis of a separate hyperinsulinism cohort and population sequencing data did not support *UCP2* variants as a monogenic cause; functional alteration alone was insufficient when frequency and segregation were inconsistent with the proposed disease [67]. This negative genetic evidence strengthens the case for cautious diagnostic interpretation rather than merely describing the relationship as unreplicated.

Because cytosolic NADH cannot cross the inner mitochondrial membrane directly, the malate-aspartate and glycerol-phosphate shuttles transfer reducing equivalents into mitochondria. Their disruption reduces glucose-induced oxidative metabolism and insulin secretion, although isolated defects in individual shuttle carriers are not established as a consistent monogenic diabetes phenotype [64,65].

### Evidence Calibration in Mitochondrial Disease

Biallelic defects in *MPC1* and selected SLC25-family genes cause severe multisystem metabolic disease in which dysglycaemia may occur, while consistent isolated diabetes is uncommon [62,63,64]. Evidence for glucose mechanisms involving MCU/NCLX and other carriers remains predominantly experimental, and human genetic data do not support UCP2 as a cause of congenital hyperinsulinism [67].

In type 2 diabetes, acquired mitochondrial dysfunction is more firmly established. Glucotoxicity, lipotoxicity, oxidative stress, altered organelle dynamics, and impaired calcium handling reduce ATP generation and metabolic amplification [50,55,61,66,74]. Because the same transport systems operate in many organs, future therapies will require β-cell targeting or pathway-selective modulation to avoid systemic toxicity.

## 9. Human Genetic Evidence and Clinical Phenotypes

A clinically useful classification separates genes that cause reproducible Mendelian dysglycaemia from loci that alter susceptibility and from experimental candidates [13,26,87]. This distinction determines diagnostic testing, variant interpretation, counselling, and therapeutic actionability. Table 4 summarizes representative pathways by human evidence category and clinical utility.

## 10. Acquired Channel and Transporter Dysfunction in Type 2 Diabetes

Acquired channel and transporter changes in type 2 diabetes can initiate dysfunction, compensate for increased demand, or follow established hyperglycemia. Cross-sectional expression differences in diabetic donor islets cannot determine this sequence. Donor age, treatment, disease duration, islet composition, isolation injury, and loss of cell identity are additional explanations. A mechanistic claim therefore requires the direction of change in current or transport, its temporal relation to secretory failure, and evidence that correction restores an appropriate physiological response [50,55,61,74].

Human *SLC30A8* protective alleles anchor susceptibility before disease, whereas K_v_2.1 clustering rescue and *VDAC1* inhibition identify reversible dysfunction in diabetic human cells [23,24,38,61]. Neither rescue establishes the initiating lesion. Altered SERCA expression or mitochondrial Ca^2+^ signals may instead reflect stress or compensation; current density, surface protein, ATP responses, compartmental Ca^2+^ and secretion help distinguish these possibilities.

### Disease Stage and Peripheral Insulin Resistance

Reduced insulin-stimulated GLUT4 trafficking increases secretory demand. Early β-cell adaptation can increase secretion; later persistent demand, ectopic lipid exposure, and organelle stress can reduce reserve and disrupt pulsatility [50]. A reduction in an outward current may initially compensate by increasing excitability yet become maladaptive if sustained depolarization drives Ca^2+^ loading or channel inactivation. Conversely, partial mitochondrial uncoupling may reduce oxidative stress while reducing ATP-dependent triggering [66]. These possibilities explain why the same intervention may help at one stage and harm at another; they are mechanistic hypotheses that require longitudinal validation.

Useful causal tests include acute and chronic perturbation in primary human islets, isogenic correction versus introduction of the same variant, and rescue of electrical and secretory function at comparable glucose exposure. Rescue should preserve pulsatility and stress tolerance, not simply raise insulin release. A defect persisting after correction of hyperglycaemia is different from one that reverses with metabolic rest, although both may be therapeutically relevant. Human genetics can provide temporal anchoring but is limited by pleiotropy and unresolved regulatory loci. Longitudinal clinical metabolic testing can relate secretion to insulin sensitivity, whereas repeated native human pancreatic sampling is rarely feasible. None of these approaches alone establishes the full sequence of type 2 diabetes.

## 11. Precision Medicine and Therapeutic Translation

### 11.1. From Molecular Diagnosis to Therapy

The strongest current example of precision therapy remains K_ATP_-channel neonatal diabetes, in which a molecular result changes drug class and often removes the need for insulin [6,7,8,9,10,72]. Genomic diagnosis also clarifies surveillance and family counselling in syndromic transporter disease, although most molecular subtypes still lack a targeted therapy [13,87]. Precision medicine should therefore be defined by demonstrable clinical utility, not by sequencing alone.

### 11.2. Functional Genomics and Variant Interpretation

Rare variants require assessment of segregation, population frequency, phenotype, and validated function; a variant of uncertain significance (VUS) should not independently determine targeted treatment [13,26,87]. Patch-clamp experiments can separate gating from surface-channel number, but heterologous cells may lack β-cell lipids, auxiliary proteins, and metabolic feedback. Standardized assays need benign and pathogenic controls, reproducibility, and a demonstrated relation between the readout and the disease mechanism. Table 5 compares the biological question each platform can answer with its limitations and realistic clinical role.

Single-cell and spatial transcriptomics resolve cell identity and anatomical expression, but mRNA abundance does not measure surface localization, open probability, current density, or transporter flux. Dissociation stress, dropout, donor variation, spatial resolution, and mixed-cell signals can distort apparent differences. Transcriptomic nominations should be tested with protein localization, electrophysiology or transport assays, and functional perturbation. These platforms are useful for prioritizing mechanisms and identifying cellular context, not for deciding that a patient needs channel blockade on the basis of expression alone.

### 11.3. Stem Cells, Organoids, and Genome Engineering

Induced pluripotent stem-cell (iPSC)-derived β-cells allow patient variants to be compared with isogenic corrected controls [90]. Their best-established use here is disease modelling and drug testing: electrophysiological maturity, glucose thresholds, metabolic coupling, cell composition, and batch variability may differ from adult primary human islets. Organoids can add vascular or immune context but do not automatically reproduce native innervation, perfusion, or whole-body insulin resistance [88,89]. A corrected-cell rescue must therefore be replicated across clones and differentiation batches and, where possible, benchmarked against primary human function.

Model limitations should not obscure progress in maturation. Benchmarking studies have generated stem-cell-derived islets with adult-like electrophysiology and biphasic secretion, despite remaining differences in glycolytic and mitochondrial metabolism [92]. Thus, maturity is protocol- and endpoint-dependent: neither uniform immaturity nor equivalence to native adult human islets should be assumed.

Human stem-cell-derived islet transplantation studies establish investigational replacement as a real clinical platform in selected diabetes populations [93,94], not routine gene-corrected therapy for *KCNJ11*-, *ABCC8*-, or transporter-related disease. CRISPR nuclease, base, and prime editing remain predominantly experimental for correction of these disorders [90,91]. In vivo delivery must reach the causal tissue at sufficient efficiency; dominant gain-of-function variants may require allele-selective correction or silencing. Ex vivo editing permits clone selection but still requires assessment of off-target and on-target structural changes, clonal selection, residual proliferative cells, immune responses, engraftment, and durable physiological regulation. An intervention confined to β-cells may not correct neurological disease or a hepatic, renal, or intestinal transport defect.

### 11.4. Therapeutic Opportunities and Constraints

Current and emerging mechanism-based therapeutic strategies are summarised in Table 6.

Tissue specificity is the central translational challenge. Most channels and carriers are shared by islets, brain, heart, kidney, intestine, or muscle, so systemic modulation can produce predictable off-target toxicity. Structure-guided ligands, targeted delivery, and disease-state biomarkers may widen the therapeutic window, but human physiological evidence must precede claims of precision benefit.

### 11.5. Equity and Implementation

Precision diagnostics can widen disparities when genomic testing, functional interpretation, and targeted treatment remain concentrated in a few centres. Underrepresentation of diverse ancestries in reference databases increases uncertainty and can reduce diagnostic yield. Sustainable implementation requires multidisciplinary teams, genetic counselling, robust data governance, validated functional networks, and mechanisms for periodic reinterpretation as knowledge changes. Table 7 aligns the main barriers with practical research priorities.

### 11.6. Unresolved Priorities and Criteria for Translation

Genotype–phenotype heterogeneity requires explanation at the level of variant function, allele dosage, trafficking, background genetic modifiers, developmental timing, and tissue expression. A shared gene name is insufficient to predict sulfonylurea response, neurological benefit, or long-term risk. Deeply phenotyped longitudinal cohorts linked to calibrated function are needed to determine which measurements improve clinical decisions beyond the molecular diagnosis itself.

Association-to-target translation requires identification of the effector gene and causal direction, replication in primary human tissue, and evidence that partial pharmacological modulation reproduces a beneficial human genetic effect. Lifelong loss of function is not equivalent to inhibition after diabetes develops. The appropriate direction and timing of modulation should be defined using baseline electrical and metabolic state, compartment-specific readouts, insulin sensitivity, and acute versus chronic exposure. Both excess and insufficient mitochondrial Ca^2+^ or membrane excitability can be harmful.

A candidate intervention should demonstrate target-tissue exposure, on-target function, preserved counter-regulation, and acceptable neurological and cardiovascular safety. Cross-donor replication and chronic studies should test durability and stress tolerance before patient trials.

## 12. Conclusions

Ion channels and transporters coordinate electrical triggering, metabolic amplification, organellar ion balance and inter-organ glucose flux. K_ATP_-related treatment and tissue-selective transporter care demonstrate the clinical value of molecular diagnosis. Priorities are to explain variant-specific heterogeneity with calibrated human functional assays, resolve causal versus compensatory remodelling through temporal and rescue experiments, and establish the direction, timing and tissue selectivity of proposed interventions.

## Figures and Tables

**Figure 1:**
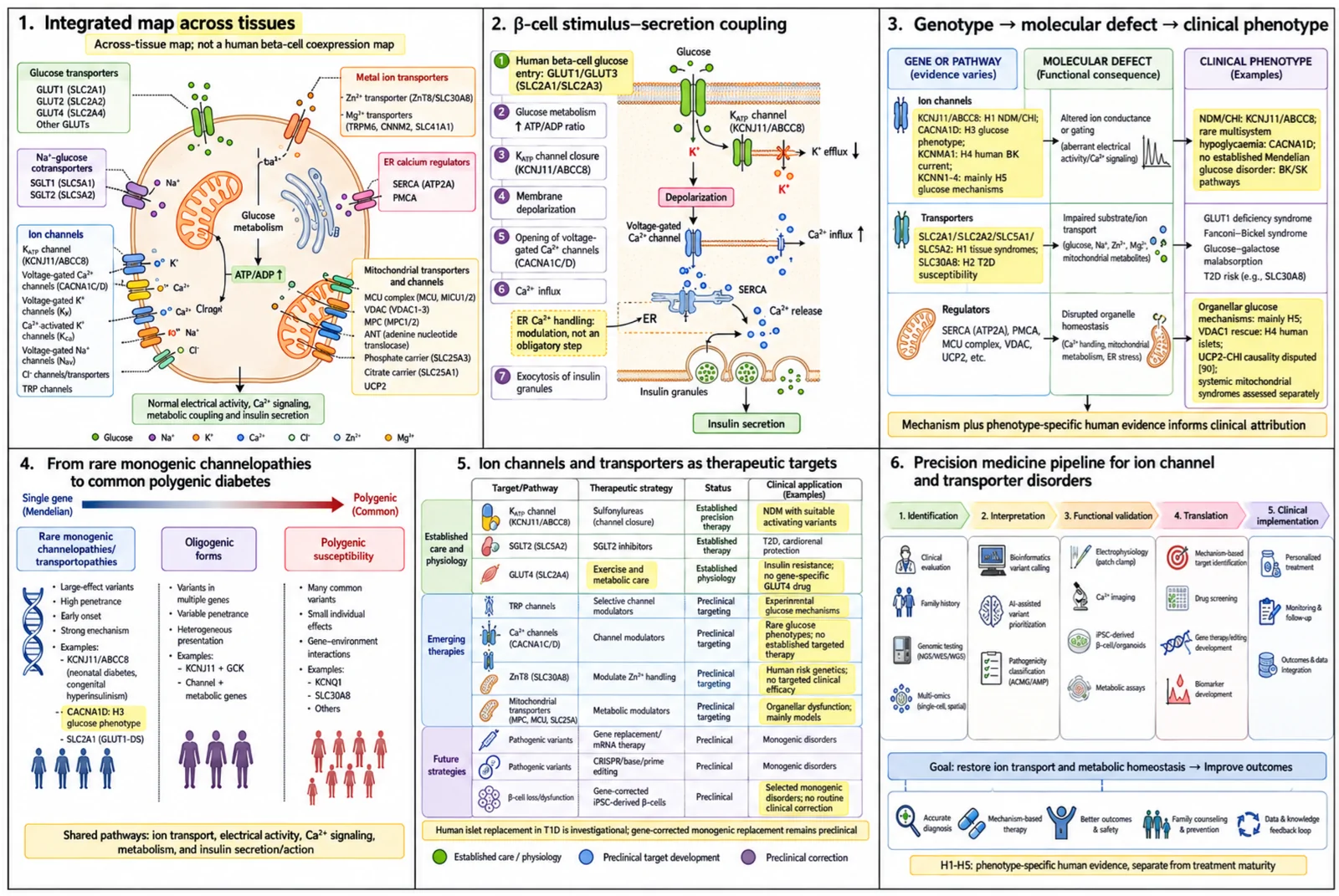
Integrated ion-channel and transporter framework. Subfigure (**1**) maps pathways across tissues, rather than human β-cell coexpression. Subfigure (**2**) shows human β-cell GLUT1/GLUT3 entry and electrical triggering; ER Ca^2+^ handling is modulatory. Subfigures (**3**,**4**) distinguish phenotype-specific human evidence from experimental glucose mechanisms. Subfigure (**5**) identifies intervention maturity separately from H1–H5, including established physiology without a gene-specific GLUT4 therapy. Subfigure (**6**) shows the translation pipeline. H1–H5 are defined in the subsection “Literature Scope and Evidence Assessment”. In (**2**), arrows show the sequence from glucose metabolism to insulin exocytosis. In (**3**), arrows link molecular defects to clinical phenotypes. In (**5**), the color key distinguishes established care or physiology, preclinical target development, and preclinical correction. Yellow highlights identify revised annotations in the current artwork. NDM, neonatal diabetes; CHI, congenital hyperinsulinism; T1D/T2D, type 1/type 2 diabetes; ER, endoplasmic reticulum.

**Table 1:** Functional integration across the β-cell secretory cycle. This phase map is conceptual and does not prescribe a universal human β-cell waveform.

Phase	Main Currents or Fluxes	Coupled Effect	Interpretative Limit
Low glucose	K_ATP_ plus background K^+^; ionic gradients maintained by pumps	Negative voltage, lower input resistance; restraint of Ca^2+^ entry	Resting voltage is not identical to K^+^ equilibrium potential
Recruitment of firing	Reduced K_ATP_; inward currents including available Na_V_/Ca_V_3.x	Higher input resistance and threshold crossing	Donor, species and inactivation state matter
Action potential and release	Ca_V_1.x/Ca_V_2.1 influx; Na_V_ where available; K_v_/BK feedback	Local Ca^2+^ triggers granule fusion; outward currents limit loading	Global Ca^2+^ amplitude does not measure release-site coupling
Sustained stimulation	Metabolic amplification; ER cycling; MCU uptake and NCLX extrusion	Granule recruitment and oxidative support of ATP	Amplification is distinct from increased Ca^2+^ triggering
Network and recovery	Cx36 current sharing; Ca^2+^ extrusion; ATP-consuming pumps	Coordination and restoration between stimuli	Isolated cells omit network context
Chronic stress	Altered trafficking, mitochondrial ATP, ER Ca^2+^ and identity	Possible compensation, then loss of reserve	Temporal and rescue evidence needed for causality

K_ATP_, ATP-sensitive potassium; Ca_V_/Na_V_/K_v_, voltage-gated calcium/sodium/potassium channels; Cx36, connexin 36; MCU, mitochondrial calcium uniporter; NCLX, mitochondrial sodium/calcium exchanger; ER, endoplasmic reticulum.

**Table 2:** Major ion channels and transporters involved in glucose homeostasis.

System	Representative Genes and Proteins	Principal Functional Evidence	Integrated Role	Human Evidence and Limitation
K_ATP_	*KCNJ11*/Kir6.2; *ABCC8*/SUR1	Human genetics; primary human β-cells; structural/expression systems	Nucleotide sensing; threshold and input resistance	H1 NDM/CHI glucose phenotypes [1,6,7,8,9,10,11,12,13]
Voltage-gated Ca^2+^	*CACNA1C*/Ca_V_1.2; *CACNA1D*/Ca_V_1.3; *CACNA1A*/Ca_V_2.1; *CACNA1H*/Ca_V_3.2	Primary human islets and β-cells; subtype-specific cellular experiments	Firing, Ca^2+^ microdomains and exocytosis are distinct endpoints	H4 human β-cell Ca2^+^ currents; H1 *CACNA1C* Timothy systemic syndrome; H3 *CACNA1D* hyperinsulinaemic glucose phenotype [1,32,33,34,35]
Voltage-gated K^+^	*KCNB1*/K_v_2.1; *KCNQ1*/K_v_7.1; *KCNH2*/K_v_11.1; *KCNH6*/K_v_11.2	Human β-cell experiments; human long-QT cohorts; rodent/cell models	Repolarization; K_v_2.1 also organizes exocytosis	H4 human K_v_2.1 exocytotic function; H2 *KCNQ1* T2D susceptibility; H1 *KCNQ1*/*KCNH2* long-QT systemic syndromes, with reported postprandial hypoglycaemia [21,22,36,37,38,39]
Background K^+^	*KCNK16*/TALK-1 (K2P16.1)	Human pedigree; transduced human islets; mouse and expression systems	Threshold, interburst voltage, and ER Ca^2+^ handling	H3 *KCNK16* diabetes pedigree; H4 transduced human-islet secretion; H5 detailed mouse ER mechanism [40,41]
Ca^2+^-activated K^+^	*KCNMA1*/KCa1.1 (BK); *KCNN1*–3/KCa2.1–2.3 (SK); *KCNN4*/KCa3.1	BK: primary human recordings; SK/KCa3.1: mainly rodents/cell models	BK: voltage and Ca^2+^; SK/KCa3.1: Ca^2+^/calmodulin feedback	H4 human BK repolarizing current; H5 detailed SK/KCa3.1 glucose mechanisms [1,42,43]
Voltage-gated Na^+^	Representative *SCN3A*/Na_V_1.3; *SCN8A*/Na_V_1.6; *SCN9A*/Na_V_1.7	Human current recordings; isoform dissection substantially mouse/expression systems	Upstroke and availability depend on membrane potential	H4 family-level physiology; isoform roles not equivalent [1,44]
Gap junction	*GJD2*/connexin 36	Primary human junction/coupling studies; rodent perturbations	Network current sharing and coordination	H4 human coupling; no established targeted treatment [30]
Chloride pathways	Representative *CFTR*; other Cl^−^ pathways discussed at family level	Human CF cohorts; mixed islet and cellular evidence	Exocrine–endocrine interactions; direct β-cell effect remains debated	H1 *CFTR* cystic fibrosis systemic syndrome; direct β-cell attribution uncertain [45]
TRP pathways	Representative *TRPM6*; other islet TRP mechanisms are family-level context	Human systemic Mg^2+^ genetics; chiefly models for direct secretion	Systemic electrolyte and cellular signalling roles differ	H1 *TRPM6* systemic hypomagnesaemia with secondary hypocalcaemia; H5 direct glucose-targeting mechanisms [46,47,48]
Facilitative glucose transport	*SLC2A1*/GLUT1; *SLC2A2*/GLUT2; *SLC2A3*/GLUT3	Human tissue/genetics; comparative rodent data	Tissue glucose delivery; human and mouse β-cell profiles differ	H1 *SLC2A1* cerebral GLUT1 deficiency and *SLC2A2* Fanconi–Bickel systemic syndrome; H4 human β-cell GLUT profile [14,15,16,27,49]
Insulin-responsive transport	*SLC2A4*/GLUT4	Human metabolic/tissue studies and mechanistic models	Insulin/contraction-dependent peripheral uptake	H4 physiology; acquired insulin resistance [50]
Sodium–glucose transport	*SLC5A1*/SGLT1; *SLC5A2*/SGLT2	Human genetics; human drug trials	Intestinal uptake and renal reabsorption, respectively	H1 *SLC5A1* glucose–galactose malabsorption and *SLC5A2* renal glucosuria; established SGLT2 pharmacology is separate [17,18,19,20,51,52]
Granule Zn^2+^	*SLC30A8*/ZnT8	Human common/rare variants; mouse and cellular granule work	Granule Zn^2+^ handling; variant direction matters	H2 T2D susceptibility and protective allelic series [23,24,53,54]
Mg^2+^ transport	*TRPM6*; *CNNM2*; *SLC41A1*	Systemic human electrolyte evidence; direct β-cell evidence limited	Free Mg^2+^, Mg-nucleotides, and systemic balance differ	H1 *TRPM6* systemic electrolyte disease; *CNNM2/SLC41A1* not assigned as primary diabetes causes [46,47,48]
ER and plasma-membrane Ca^2+^	*ATP2A2*/SERCA2b; *ATP2A3*/SERCA3; *ATP2B1*–4/PMCA	Detailed causal studies, mainly on mouse β-cells and cell models	Compartmental storage and extrusion	H5 for proposed diabetes interventions [55,56,57,58]
Mitochondrial Ca^2+^	MCU, *MICU1*/2, *SMDT1*/EMRE; *SLC8B1*/NCLX	Rodent/cellular perturbations; selected human-islet work	Uptake–extrusion balance; metabolism and stress	H5 glucose targeting; no routine isolated diabetes attribution [28,29,59,60]
Mitochondrial metabolites	*VDAC1*–3; *MPC1*/2; ANT (*SLC25A4*/5/6/31); *SLC25A3*; *SLC25A1*; *UCP2*	Mixed human-islet, rodent, cellular and biochemical evidence	ATP/substrate exchange and amplification; localisation matters	H4 *VDAC1* human-islet ex vivo rescue; other glucose mechanisms mainly H5. *UCP2*–CHI causality challenged by negative human genetics [61,62,63,64,65,66,67]

H1–H5 describe human evidence (the subsection “Literature Scope and Evidence Assessment”), independently of intervention maturity. BK/SK, large-/small-conductance calcium-activated potassium channels; NDM, neonatal diabetes mellitus; CHI, congenital hyperinsulinism; ANT, adenine nucleotide translocase; MPC, mitochondrial pyruvate carrier; VDAC, voltage-dependent anion channel. Abbreviations: ATP, adenosine triphosphate; Ca^2+^, calcium; ER, endoplasmic reticulum; K_ATP_, ATP-sensitive potassium; Mg^2+^, magnesium; SGLT, sodium-glucose cotransporter; T2D, type 2 diabetes; Zn^2+^, zinc.

**Table 3:** Bidirectional and allelic-series effects linking molecular function to dysglycaemia.

Gene or Pathway	Gain-of-Function Consequence	Human Phenotype	Loss-of-Function Consequence	Human Phenotype	Interpretation
*KCNJ11*/Kir6.2	Increased K_ATP_ channel activity; channel remains excessively open	Neonatal diabetes, including the DEND spectrum	Reduced K_ATP_ channel activity/closure	Congenital hyperinsulinism	Opposite effects on β-cell membrane excitability produce opposite glucose phenotypes [6,7,8,13]
*ABCC8*/SUR1	Increased K_ATP_ channel activity or impaired ATP-dependent closure	Neonatal diabetes	Reduced channel activity/closure	Congenital hyperinsulinism; selected inactivating variants are also associated with later diabetes, including after an earlier hyperinsulinaemic phase [13,71]	Clinical course and measured variant function are required; later diabetes must not automatically be assigned to gain of function [9,10,11,12,13,71]
*CACNA1D/CaV1.3*	Increased Ca^2+^ influx	Hyperinsulinaemic hypoglycaemia in rare multisystem cases	Reduced Ca^2+^ influx; predicted impairment of secretion	No reproducible isolated dysglycaemia phenotype established	Activating-variant evidence is limited to rare multisystem diseases [33,34]
*SLC30A8*/ZnT8	Altered Zn^2+^ handling; phenotype depends on variant	Increased T2D susceptibility for selected variants	Some protein-truncating variants appear protective against T2D	Reduced diabetes susceptibility	Illustrates that transporter variation can produce non-classical genotype–phenotype relationships [23,24]

DEND, developmental delay, epilepsy and neonatal diabetes; K_ATP_, ATP-sensitive potassium channel; T2D, type 2 diabetes.

**Table 4:** Human evidence categories and clinical utility of representative ion-channel and transporter pathways.

Human Category and Phenotype	Gene or Pathway	Genetic and Functional Basis	Diagnostic Utility	Treatment Evidence and Boundary
H1 NDM/CHI glucose phenotypes	*KCNJ11*; *ABCC8*	Replicated NDM/CHI; human cohorts and functional gating/trafficking	High diagnostic utility in appropriate phenotype	Sulfonylurea transfer for suitable activating variants; CHI treatment depends on residual function [6,7,8,9,10,11,12,13]
H1 cerebral or hepatic/renal transport syndromes	*SLC2A1*; *SLC2A2*	Established tissue-selective transport syndromes	Neurological or hepatic/renal metabolic diagnosis	Ketogenic therapy for GLUT1 deficiency; nutritional/renal care for Fanconi–Bickel [14,15,16,79]
H1 intestinal malabsorption or renal glucosuria	*SLC5A1*; *SLC5A2*	Human malabsorption or renal glucosuria	Diagnostic utility for corresponding phenotype	Dietary sugar restriction or supportive care; SGLT2 drugs treat other indicated populations [17,18,19,20]
H1 exercise-induced hypoglycaemia or syndromic diabetes	*SLC16A1*; *SLC19A2*; *SLC29A3*	Replicated regulatory or recessive syndromes	Molecular diagnosis and tailored surveillance	Exercise-related prevention; thiamine in *SLC19A2*; multisystem diabetes care in *SLC29A3* [80,81,82]
H1 systemic syndrome	*CACNA1C*	Established Timothy syndrome; intermittent hypoglycaemia reported within a multisystem phenotype	Syndrome-specific cardiac and developmental diagnosis	No established genotype-directed glucose therapy [32]
H1 systemic syndrome	*CFTR*; *TRPM6*	Established CF/electrolyte disease; dysglycaemia can be secondary	Syndrome-specific screening or electrolyte assessment	Insulin in CF-related diabetes; Mg^2+^ replacement for deficiency, not proof of direct β-cell correction [45,46,47,48]
H2 T2D susceptibility or protective allelic series	*KCNQ1*; *SLC30A8*	Replicated T2D associations/allelic series; physiological support varies	Not sufficient for monogenic diagnosis from a common allele	No validated genotype-selected channel/ZnT8 drug; effector and dosing questions remain [21,22,23,24]
H3 glucose phenotype	*CACNA1D*; *KCNK16*	Rare multisystem cases or limited pedigrees; functional support	Specialist interpretation; independent glucose-phenotype replication needed	No established genotype-directed glucose treatment; cardiac/neural context important [33,34,40]
H4 measured human cellular functions	Human β-cell Ca_V_/Na_V_/BK currents; K_v_2.1; Cx36; *VDAC1* rescue	Primary human functional evidence without a uniform Mendelian glucose disorder	Mechanistic interpretation; not a diagnostic gene list	No established selective glucose therapy; functional rescue is not a clinical trial [1,30,38,61]
H5 glucose mechanism	MCU/NCLX, PMCA, *UCP2*, selected mitochondrial carriers	Predominantly model perturbation. *UCP2*–hyperinsulinism causality is challenged by negative cohort/population genetic evidence [67]	Do not label model necessity as isolated human diabetes causality	Preclinical targeting; established multisystem mitochondrial diagnoses remain separate [28,29,57,58,59,60,61,62,63,64,65,66,67,86]

H1–H5 are phenotype-specific human-evidence categories defined in the subsection “Literature Scope and Evidence Assessment”, not treatment grades. NDM, neonatal diabetes mellitus; CHI, congenital hyperinsulinism; CF, cystic fibrosis; T2D, type 2 diabetes. Established systemic syndromes and secondary glucose effects are identified separately from primary dysglycaemia. Functional models, diagnostic utility and treatment evidence are reported independently.

**Table 5:** Technologies enabling precision diagnosis and therapeutic development.

Platform	Best Question	Source	Main Limitation	Clinical or Translational Role
Panel/exome/genome sequencing	Which variant or structural defect is present?	Human DNA; familial segregation where available	Coverage, mosaicism, noncoding interpretation, incidental findings	Established molecular diagnosis for validated genes; not proof of function
Long-read sequencing	Does a complex region or structural variant explain disease?	Long DNA molecules	Cost, error calibration, interpretation and validation	Specialist diagnostic adjunct; utility depends on indication
GWAS and allelic series	Which locus or dosage direction affects risk?	Human population genetics	Linkage disequilibrium, ancestry, pleiotropy; gene assignment	Target prioritisation; common risk allele is not monogenic diagnosis
Single-cell/spatial transcriptomics	Which cell type or location expresses a candidate?	Primary tissue or model transcriptomes	mRNA is not surface protein, gating or flux; dropout and tissue processing	Discovery and context; requires functional confirmation
Patch clamp and transport assays	Is gating, trafficking or flux altered?	Primary human cells, stem-cell cells or heterologous systems	Dialysis, auxiliary proteins, maturation and expression artefacts	Variant evidence only when the assay is calibrated and mechanism-relevant
Ca^2+^ imaging and metabolic assays	Which compartment or coupling step is affected?	Human islets or experimental systems	Indicator loading, compartment targeting, temporal resolution; total versus free ions	Mechanistic validation; should be linked to current and secretion
iPSC-derived β-cells	Does an isogenic variant change cell function?	Patient-derived or engineered human cells	Clone/batch effects; immature electrical/metabolic phenotype	Disease modelling and screening; not automatically native adult physiology
Organoids	How do cellular interactions modify phenotype?	Engineered multicellular tissue	Incomplete perfusion, innervation and systemic context	Experimental context and maturation studies [88,89]
CRISPR/base/prime editing	Does correction rescue a defined defect?	Usually cultured cells or preclinical grafts	Delivery, off-target/on-target damage, mosaicism, clonal selection, durability	Causal testing; monogenic corrective treatment remains experimental [90,91]
Cryo-EM and high-throughput assays	How does structure/variant diversity affect function?	Purified proteins; expression screens	Static state, assay context and off-target selectivity	Drug/variant prioritisation; needs physiological validation
AI-assisted interpretation	Which variants warrant expert review?	Computational models	Training bias, data leakage, calibration, uncertain generalisation	Prioritisation adjunct; never an independent pathogenic diagnosis

GWAS, genome-wide association study; iPSC, induced pluripotent stem cell; CRISPR, clustered regularly interspaced short palindromic repeats; Cryo-EM, cryogenic electron microscopy; AI, artificial intelligence.

**Table 6:** Mechanism-based therapeutic strategies and translational constraints.

Maturity	Genotype or Molecular Defect	Phenotype/Context	Intervention	Human Evidence	Translation Limit
Established	*KCNJ11*/*ABCC8* activation; excess K_ATP_ opening	NDM ± neurological phenotype	Sulfonylureas in suitable genotypes	Human transfer studies and long-term cohorts [6,7,8,9,10]	Variant-specific response; specialist protocol; neural benefit incomplete
Established	*KCNJ11*/*ABCC8* inactivation; reduced opening/trafficking	Focal or diffuse CHI	Diazoxide when responsive; genotype/imaging-guided management	Human clinical cohorts [11,12,13]	Severe loss often unresponsive; the genotype does not replace clinical assessment
Established	*SLC2A1* loss; cerebral glucose shortage	GLUT1 deficiency	Ketogenic therapy	Disease-specific human clinical evidence [14,15,79]	Alternative fuel provision; not correction of transporter
Established	*SLC5A1* loss; intestinal sugar uptake failure	Glucose–galactose malabsorption	Restrict glucose/galactose; tailored nutrition	Human disease management [17,18]	Nutritional specialist oversight
Established	*SLC19A2* loss; impaired thiamine transport	Thiamine-responsive diabetes syndrome	Pharmacological thiamine; insulin if required	Human syndrome evidence [81]	Response varies; hearing disease may persist
Established	Renal SGLT2 pharmacological inhibition; not selected by *SLC5A2* genotype	Indicated T2D/cardiorenal populations	SGLT2 inhibitor	Randomised human outcomes [51,52]	Indication-specific; ketoacidosis/volume risks; not genetic correction
Established supportive	*TRPM6*-related or acquired Mg^2+^ deficiency	Electrolyte depletion	Mg^2+^ replacement	Human electrolyte disease [46,47]	Not a validated β-cell-specific diabetes intervention
Established physiology	Acquired insulin resistance/GLUT4 trafficking	Peripheral glucose disposal defect	Exercise and established metabolic care	Human physiology; multiple pathways [50]	Not a gene-specific GLUT4 drug
Investigational in humans	β-cell loss; no monogenic gene correction established	Selected T1D transplant populations	Stem-cell-derived islet replacement	Human studies [93,94]	Immune protection, engraftment, durability; not evidence for gene-corrected monogenic use
Preclinical targeting	*SLC30A8* dosage/variant effects; granule Zn^2+^	T2D susceptibility	Proposed ZnT8 modulation	Human protective genetics; no cited targeted patient efficacy trial [23,24]	Lifetime genetic loss differs from timed drug inhibition
Preclinical targeting	*KCNK16* activation; background current	Candidate monogenic/complex glucose dysfunction	Proposed TALK-1 modulation	Limited human genetics and ex vivo function [40]	No validated selective patient therapy
Preclinical targeting	K_v_2.1 scaffold; *VDAC1* localisation	Acquired β-cell dysfunction	Preserve clustering or inhibit pathological surface *VDAC1*	Human ex vivo rescue [38,61]	No demonstrated clinical efficacy or systemic therapeutic window
Preclinical targeting	SERCA, MPC, MCU/NCLX and related transport	ER/mitochondrial coupling dysfunction	Compartment-selective modulation	Mainly rodent/cellular intervention [28,29,55,56,57,58,59,60,61,62,63,64,65,66]	Direction, timing, tissue selectivity and chronic safety unresolved
Preclinical correction	Defined pathogenic channel/transporter variants	Selected monogenic disorders	Gene/RNA approaches or gene-corrected stem-cell islets	Cell/model rescue; no routine corrective treatment demonstrated here [90,91]	Causal tissue, delivery, genomic integrity, immunity and long-term safety

Maturity concerns the specified intervention and population. NDM, neonatal diabetes mellitus; CHI, congenital hyperinsulinism; T1D/T2D, type 1/type 2 diabetes; ER, endoplasmic reticulum; MPC, mitochondrial pyruvate carrier; MCU, mitochondrial calcium uniporter; NCLX, mitochondrial sodium/calcium exchanger.

**Table 7:** Priority barriers and research actions for precision medicine.

Challenge	Current Limitation	Research Priority	Practical Approach	Expected Impact
Access to genomic diagnosis	Testing and specialist interpretation remain uneven	Embed sequencing and reanalysis in diabetes pathways	Regional genomic services and multidisciplinary review	Earlier, more equitable molecular diagnosis
Variants of uncertain significance	Prediction often lacks functional evidence	Develop calibrated high-throughput assays	Patch clamp, transport assays, calcium imaging, and isogenic stem-cell models	Stronger and faster variant classification
Genotype–phenotype variability	The same gene can produce divergent phenotypes	Build deeply phenotyped longitudinal cohorts	International registries with harmonized outcomes	Improved prognosis and treatment selection
Tissue specificity	Targets are widely expressed across organs	Develop β-cell- or organ-selective delivery	Targeted vectors, prodrugs, and state-dependent ligands	Wider therapeutic window and less toxicity
Mitochondrial complexity	Perturbations affect multiple pathways and organs	Define disease-specific metabolic signatures	Multi-omics linked to quantitative functional phenotyping	More rational selection of mitochondrial targets
Population diversity	Reference datasets underrepresent many ancestries	Expand globally representative genomic resources	International recruitment and ancestry-aware interpretation	Higher diagnostic accuracy and equity
Gene and cell therapy safety	Delivery, genomic integrity, immunity, and durability remain uncertain	Establish long-term safety and efficacy	Prospective trials, registries, and standardized surveillance	Responsible translation of corrective therapies
Data governance	Reanalysis and incidental findings create operational challenges	Create transparent consent and governance frameworks	Secure data systems, expert review, and scheduled reinterpretation	Sustainable clinical implementation

Implementation priorities do not imply that proposed corrective treatments are clinically established.

## Data Availability

No new experimental data were generated. The search audit materials supporting this narrative update are provided in Supplementary Search Methods S1 and Supplementary Search Audit S2.

## Supplementary Materials

The following supporting information can be downloaded at https://www.mdpi.com/article/10.3390/cells15212030/s1. Supplementary Search Methods S1 provides exact PubMed queries, translations, retrieval parameters, ordered block-specific PMID lists, selection details and limitations. Supplementary Search Audit S2 (separate ZIP archive) contains the original search responses, XML exports, selection records and code, with derived ordered PMID files and a checksum manifest.

## Abbreviations

ANT, adenine nucleotide translocase; CHI, congenital hyperinsulinism; CRISPR, clustered regularly interspaced short palindromic repeats; Cx36, connexin 36; DEND, developmental delay, epilepsy and neonatal diabetes; EMRE, essential MCU regulator; ER, endoplasmic reticulum; GLUT, facilitative glucose transporter; GSIS, glucose-stimulated insulin secretion; iPSC, induced pluripotent stem cell; K_ATP_, ATP-sensitive potassium channel; MCU, mitochondrial calcium uniporter; MPC, mitochondrial pyruvate carrier; NCLX, mitochondrial Na^+^/Ca^2+^ exchanger; NDM, neonatal diabetes mellitus; PMCA, plasma-membrane Ca^2+^-ATPase; PNDM, permanent neonatal diabetes mellitus; SERCA, sarco/endoplasmic-reticulum Ca^2+^-ATPase; SGLT, sodium-glucose cotransporter; T2D, type 2 diabetes; TNDM, transient neonatal diabetes mellitus; VDAC, voltage-dependent anion channel; VUS, variant of uncertain significance.
