# Supplementary material for: Ion Channels and Transporters in Disorders of Glucose Homeostasis: From Molecular Mechanisms to Precision Medicine
Source: Cells. 2026 Nov 6;15(21):2030. doi: 10.3390/cells15212030 (PMC13762379; doi:10.3390/cells15212030)
Supplement: Supplementary file 1 [file cells-15-02030-s001.zip › Cells_Supplementary_Search_Audit_S2 2/Cells_Supplementary_Search_Methods_S1.pdf]

# Supplementary Search Methods S1

Structured PubMed update for Ion Channels and Transporters in Disorders of Glucose Homeostasis

## Purpose and scope

This supplement documents a newly executed search on 23 October 2026. It replaces an unrecorded historical search description; it does not reconstruct the original authors' search. PubMed (NCBI, including MEDLINE) was queried directly through the NCBI Entrez ESearch and EFetch interfaces. Publisher and PubMed Central full text were used selectively for source checking. No other bibliographic database was searched.

The final search specification used title/abstract gene and pathway terms, with Humans MeSH additionally used in S4, and a publication-date range from 1 January 1800 through 23 October 2026. No language, human-only, publication-type or open-access filter was applied globally. Search blocks intentionally overlap. PubMed indexing, ranking and metadata can change, so the saved translations, identifiers and XML exports are the reproducibility record for this execution. The original records and derived audit files are supplied in Supplementary Search Audit S2.

## Retrieval and selection

Each Entrez ESearch request specified sort=relevance and retmax=60, with retstart omitted; all eight responses recorded retstart=0 and retmax=60. The 60 returned identifiers per block were retained in their original order before PMID-based deduplication. The 480 block-specific records represented 467 unique identifiers: 465 journal records and two book/database entries. AI-assisted title assessment prioritised 67 journal records, 63 with indexed abstracts, for closer assessment. Accessible full text was consulted selectively for key mechanistic claims, including Cx36 rescue, calcium-channel organisation and disputed UCP2–hyperinsulinism causality. Priorities were primary human genetic, human-islet and interventional evidence, variant-function studies, contradictory findings and clinical guidance; reviews supplied context, and relevant foundational references were retained. H.D. and K.H. verified the AI-assisted screening, key source interpretations and final H1–H5 assignments. This was not independent duplicate screening; no formal risk-of-bias assessment or meta-analysis was performed. Single-database coverage and relevance-ranked caps leave potentially relevant records unretrieved, including recent publications within the date range; the update is not comprehensive through the search date.

The total of 17,706 block-specific matches is a sum of overlapping counts, not a count of unique studies identified or screened. Only the capped retrievals were reviewed. Deduplication used PMID, not title or DOI; multiple records describing related work can therefore remain. Two retrieved book/database records (PMIDs 35442606 and 25905200) were not treated as primary journal evidence. Four of the 67 prioritised journal records lacked an indexed abstract; their record-level information or accessible full text was used where relevant. This is an audit of narrative prioritisation, not a PRISMA eligibility flow.

Selection questions were: Does the report materially clarify a human causal gene–phenotype relationship, physiological coupling, contradictory evidence, a model limitation, or an established versus experimental intervention? Repeated small reports, peripheral complication-only papers, generic reviews and studies outside the central question were generally lower priority. These were purposive priorities, not prospectively registered exclusion rules. Full text was not obtained for every prioritised record, and author verification does not constitute independent duplicate screening.

## Search blocks and exact queries

## S1 KATP

Executed UTC: 2026-10-23T10:48:56.315070+00:00. Matches: 3404. Retrieved: 60. ESearch parameters: sort=relevance; retmax=60; retstart omitted. Response: retstart=0; retmax=60. No pagination beyond these 60 returned identifiers.

Exact query: (KCNJ11[Title/Abstract] OR ABCC8[Title/Abstract] OR Kir6.2[Title/Abstract] OR SUR1[Title/Abstract] OR KATP[Title/Abstract]) AND (diabet*[Title/Abstract] OR "insulin secretion"[Title/Abstract] OR hyperinsulin*[Title/Abstract] OR "glucose homeostasis"[Title/Abstract] OR "beta cell"[Title/Abstract] OR "beta cells"[Title/Abstract] OR islet*[Title/Abstract]) AND ("1800/01/01"[Date - Publication] : "2026/10/23"[Date - Publication])

PubMed query translation: ("KCNJ11"[Title/Abstract] OR "ABCC8"[Title/Abstract] OR "Kir6.2"[Title/Abstract] OR "SUR1"[Title/Abstract] OR "KATP"[Title/Abstract]) AND ("diabet*"[Title/Abstract] OR "insulin secretion"[Title/Abstract] OR "hyperinsulin*"[Title/Abstract] OR "glucose homeostasis"[Title/Abstract] OR "beta cell"[Title/Abstract] OR "beta cells"[Title/Abstract] OR "islet*"[Title/Abstract]) AND 1800/01/01:2026/10/23[Date - Publication]

## S2 excitable channels

Executed UTC: 2026-10-23T10:49:02.201559+00:00. Matches: 651. Retrieved: 60. ESearch parameters: sort=relevance; retmax=60; retstart omitted. Response: retstart=0; retmax=60. No pagination beyond these 60 returned identifiers.

Exact query: (CACNA1D[Title/Abstract] OR CACNA1C[Title/Abstract] OR CACNA1A[Title/Abstract] OR KCNB1[Title/Abstract] OR KCNQ1[Title/Abstract] OR KCNH2[Title/Abstract] OR KCNH6[Title/Abstract] OR KCNK16[Title/Abstract] OR "TALK-1"[Title/Abstract] OR "TALK-2"[Title/Abstract] OR KCNMA1[Title/Abstract] OR KCNN4[Title/Abstract] OR "sodium channels"[Title/Abstract] OR "SK channels"[Title/Abstract]) AND (diabet*[Title/Abstract] OR "insulin secretion"[Title/Abstract] OR hyperinsulin*[Title/Abstract] OR "glucose homeostasis"[Title/Abstract] OR "beta cell"[Title/Abstract] OR "beta cells"[Title/Abstract] OR islet*[Title/Abstract]) AND ("1800/01/01"[Date - Publication] : "2026/10/23"[Date - Publication])

PubMed query translation: ("CACNA1D"[Title/Abstract] OR "CACNA1C"[Title/Abstract] OR "CACNA1A"[Title/Abstract] OR "KCNB1"[Title/Abstract] OR "KCNQ1"[Title/Abstract] OR "KCNH2"[Title/Abstract] OR "KCNH6"[Title/Abstract] OR "KCNK16"[Title/Abstract] OR "TALK-1"[Title/Abstract] OR "TALK-2"[Title/Abstract] OR "KCNMA1"[Title/Abstract] OR "KCNN4"[Title/Abstract] OR "sodium channels"[Title/Abstract] OR "SK channels"[Title/Abstract]) AND ("diabet*"[Title/Abstract] OR "insulin secretion"[Title/Abstract] OR "hyperinsulin*"[Title/Abstract] OR "glucose homeostasis"[Title/Abstract] OR "beta cell"[Title/Abstract] OR "beta cells"[Title/Abstract] OR "islet*"[Title/Abstract]) AND 1800/01/01:2026/10/23[Date - Publication]

## S3 network

Executed UTC: 2026-10-23T10:49:07.588578+00:00. Matches: 136. Retrieved: 60. ESearch parameters: sort=relevance; retmax=60; retstart omitted. Response: retstart=0; retmax=60. No pagination beyond these 60 returned identifiers.

Exact query: (GJD2[Title/Abstract] OR "connexin 36"[Title/Abstract] OR connexin36[Title/Abstract] OR Cx36[Title/Abstract] OR "metabolic amplification"[Title/Abstract]) AND (diabet*[Title/Abstract] OR "insulin secretion"[Title/Abstract] OR hyperinsulin*[Title/Abstract] OR "glucose homeostasis"[Title/Abstract] OR "beta cell"[Title/Abstract] OR "beta cells"[Title/Abstract] OR islet*[Title/Abstract]) AND ("1800/01/01"[Date - Publication] : "2026/10/23"[Date - Publication])

PubMed query translation: ("GJD2"[Title/Abstract] OR "connexin 36"[Title/Abstract] OR "connexin36"[Title/Abstract] OR "Cx36"[Title/Abstract] OR "metabolic amplification"[Title/Abstract]) AND ("diabet*"[Title/Abstract] OR "insulin secretion"[Title/Abstract] OR "hyperinsulin*"[Title/Abstract] OR "glucose homeostasis"[Title/Abstract] OR "beta cell"[Title/Abstract] OR "beta cells"[Title/Abstract] OR "islet*"[Title/Abstract]) AND 1800/01/01:2026/10/23[Date - Publication]

## S4 glucose transport

Executed UTC: 2026-10-23T10:49:13.890774+00:00. Matches: 10399. Retrieved: 60. ESearch parameters: sort=relevance; retmax=60; retstart omitted. Response: retstart=0; retmax=60. No pagination beyond these 60 returned identifiers.

Exact query: (SLC2A1[Title/Abstract] OR SLC2A2[Title/Abstract] OR SLC2A4[Title/Abstract] OR SLC5A1[Title/Abstract] OR SLC5A2[Title/Abstract] OR "SGLT2 inhibitor"[Title/Abstract] OR "SGLT2 inhibitors"[Title/Abstract] OR "GLUT1 deficiency"[Title/Abstract] OR "Fanconi-Bickel"[Title/Abstract] OR "glucose-galactose malabsorption"[Title/Abstract]) AND (human[Title/Abstract] OR patient*[Title/Abstract] OR trial[Title/Abstract] OR mutation*[Title/Abstract] OR variant*[Title/Abstract] OR "Humans"[MeSH Terms]) AND ("1800/01/01"[Date - Publication] : "2026/10/23"[Date - Publication])

PubMed query translation: ("SLC2A1"[Title/Abstract] OR "SLC2A2"[Title/Abstract] OR "SLC2A4"[Title/Abstract] OR "SLC5A1"[Title/Abstract] OR "SLC5A2"[Title/Abstract] OR "SGLT2 inhibitor"[Title/Abstract] OR "SGLT2 inhibitors"[Title/Abstract] OR "GLUT1 deficiency"[Title/Abstract] OR "Fanconi-Bickel"[Title/Abstract] OR "glucose-galactose malabsorption"[Title/Abstract]) AND ("human"[Title/Abstract] OR "patient*"[Title/Abstract] OR "trial"[Title/Abstract] OR "mutation*"[Title/Abstract] OR "variant*"[Title/Abstract] OR "Humans"[MeSH Terms]) AND 1800/01/01:2026/10/23[Date - Publication]

## S5 metals and carriers

Executed UTC: 2026-10-23T10:49:19.731338+00:00. Matches: 1000. Retrieved: 60. ESearch parameters: sort=relevance; retmax=60; retstart omitted. Response: retstart=0; retmax=60. No pagination beyond these 60 returned identifiers.

Exact query: (SLC30A8[Title/Abstract] OR ZnT8[Title/Abstract] OR TRPM6[Title/Abstract] OR CNNM2[Title/Abstract] OR SLC41A1[Title/Abstract] OR SLC16A1[Title/Abstract] OR SLC19A2[Title/Abstract] OR SLC29A3[Title/Abstract]) AND (diabet*[Title/Abstract] OR "insulin secretion"[Title/Abstract] OR hyperinsulin*[Title/Abstract] OR "glucose homeostasis"[Title/Abstract] OR "beta cell"[Title/Abstract] OR "beta cells"[Title/Abstract] OR islet*[Title/Abstract]) AND ("1800/01/01"[Date - Publication] : "2026/10/23"[Date - Publication])

PubMed query translation: ("SLC30A8"[Title/Abstract] OR "ZnT8"[Title/Abstract] OR "TRPM6"[Title/Abstract] OR "CNNM2"[Title/Abstract] OR "SLC41A1"[Title/Abstract] OR "SLC16A1"[Title/Abstract] OR "SLC19A2"[Title/Abstract] OR "SLC29A3"[Title/Abstract]) AND ("diabet*"[Title/Abstract] OR "insulin secretion"[Title/Abstract] OR

"hyperinsulin*"[Title/Abstract] OR "glucose homeostasis"[Title/Abstract] OR "beta cell"[Title/Abstract] OR "beta cells"[Title/Abstract] OR "islet*"[Title/Abstract]) AND 1800/01/01:2026/10/23[Date - Publication]

## S6 organelles

Executed UTC: 2026-10-23T10:49:25.617689+00:00. Matches: 978. Retrieved: 60. ESearch parameters: sort=relevance; retmax=60; retstart omitted. Response: retstart=0; retmax=60. No pagination beyond these 60 returned identifiers.

Exact query: (MCU[Title/Abstract] OR MICU2[Title/Abstract] OR NCLX[Title/Abstract] OR SLC8B1[Title/Abstract] OR VDAC1[Title/Abstract] OR "mitochondrial pyruvate carrier"[Title/Abstract] OR UCP2[Title/Abstract] OR SERCA2b[Title/Abstract] OR PMCA[Title/Abstract] OR SLC25A3[Title/Abstract] OR SLC25A1[Title/Abstract]) AND (diabet*[Title/Abstract] OR "insulin secretion"[Title/Abstract] OR hyperinsulin*[Title/Abstract] OR "glucose homeostasis"[Title/Abstract] OR "beta cell"[Title/Abstract] OR "beta cells"[Title/Abstract] OR islet*[Title/Abstract]) AND ("1800/01/01"[Date - Publication] : "2026/10/23"[Date - Publication])

PubMed query translation: ("MCU"[Title/Abstract] OR "MICU2"[Title/Abstract] OR "NCLX"[Title/Abstract] OR "SLC8B1"[Title/Abstract] OR "VDAC1"[Title/Abstract] OR "mitochondrial pyruvate carrier"[Title/Abstract] OR "UCP2"[Title/Abstract] OR "SERCA2b"[Title/Abstract] OR "PMCA"[Title/Abstract] OR "SLC25A3"[Title/Abstract] OR "SLC25A1"[Title/Abstract]) AND ("diabet*"[Title/Abstract] OR "insulin secretion"[Title/Abstract] OR "hyperinsulin*"[Title/Abstract] OR "glucose homeostasis"[Title/Abstract] OR "beta cell"[Title/Abstract] OR "beta cells"[Title/Abstract] OR "islet*"[Title/Abstract]) AND 1800/01/01:2026/10/23[Date - Publication]

## S7 remodelling

Executed UTC: 2026-10-23T10:49:31.192965+00:00. Matches: 106. Retrieved: 60. ESearch parameters: sort=relevance; retmax=60; retstart omitted. Response: retstart=0; retmax=60. No pagination beyond these 60 returned identifiers.

Exact query: ("human islets"[Title/Abstract] OR "human beta cells"[Title/Abstract] OR "human beta-cell"[Title/Abstract]) AND ("type 2 diabetes"[Title/Abstract] OR diabetic[Title/Abstract]) AND (electrophysiolog*[Title/Abstract] OR channel*[Title/Abstract] OR "calcium handling"[Title/Abstract] OR "insulin exocytosis"[Title/Abstract]) AND ("1800/01/01"[Date - Publication] : "2026/10/23"[Date - Publication])

PubMed query translation: ("human islets"[Title/Abstract] OR "human beta cells"[Title/Abstract] OR "human beta-cell"[Title/Abstract]) AND ("type 2 diabetes"[Title/Abstract] OR "diabetic"[Title/Abstract]) AND ("electrophysiolog*"[Title/Abstract] OR "channel*"[Title/Abstract] OR "calcium handling"[Title/Abstract] OR "insulin exocytosis"[Title/Abstract]) AND 1800/01/01:2026/10/23[Date - Publication]

## S8 translation

Executed UTC: 2026-10-23T10:49:37.174949+00:00. Matches: 1032. Retrieved: 60. ESearch parameters: sort=relevance; retmax=60; retstart omitted. Response: retstart=0; retmax=60. No pagination beyond these 60 returned identifiers.

Exact query: ("monogenic diabetes"[Title/Abstract] OR "neonatal diabetes"[Title/Abstract] OR "congenital hyperinsulinism"[Title/Abstract] OR "stem cell-derived islets"[Title/Abstract] OR "stem-cell-derived islets"[Title/Abstract]) AND ("precision medicine"[Title/Abstract] OR sulfonylurea*[Title/Abstract] OR

diazoxide[Title/Abstract] OR CRISPR[Title/Abstract] OR "gene editing"[Title/Abstract] OR transplant*[Title/Abstract] OR guideline*[Title/Abstract]) AND ("1800/01/01"[Date - Publication] : "2026/10/23"[Date - Publication])

PubMed query translation: ("monogenic diabetes"[Title/Abstract] OR "neonatal diabetes"[Title/Abstract] OR "congenital hyperinsulinism"[Title/Abstract] OR "stem cell derived islets"[Title/Abstract] OR "stem cell derived islets"[Title/Abstract]) AND ("precision medicine"[Title/Abstract] OR "sulfonylurea*"[Title/Abstract] OR "diazoxide"[Title/Abstract] OR "CRISPR"[Title/Abstract] OR "gene editing"[Title/Abstract] OR "transplant*"[Title/Abstract] OR "guideline*"[Title/Abstract]) AND 1800/01/01:2026/10/23[Date - Publication]

## Sources added to the revised manuscript

Reference 88. Restoring connexin-36 function in diabetogenic environments precludes mouse and human islet dysfunction. PMID 37578890. DOI 10.1113/JP282114. Functional source and translation boundary are stated in the revised main text.

Reference 89. Ca2+ channel clustering with insulin-containing granules is disturbed in type 2 diabetes. PMID 28481223. DOI 10.1172/JCI88491. Functional source and translation boundary are stated in the revised main text.

Reference 90. Analysis of large-scale sequencing cohorts does not support the role of variants in UCP2 as a cause of hyperinsulinaemic hypoglycaemia. PMID 28681398. DOI 10.1002/humu.23289. Functional source and translation boundary are stated in the revised main text.

Reference 91. Functional, metabolic and transcriptional maturation of human pancreatic islets derived from stem cells. PMID 35241836. DOI 10.1038/s41587-022-01219-z. Functional source and translation boundary are stated in the revised main text.

Reference 92. Loss of electrical β-cell to δ-cell coupling underlies impaired hypoglycaemia-induced glucagon secretion in type-1 diabetes. PMID 39313541. DOI 10.1038/s42255-024-01139-z. Functional source and translation boundary are stated in the revised main text.

Reference 93. Zinc accumulation-induced integrated stress response triggers β-cell identity loss. PMID 41606169. DOI 10.1038/s41422-026-01222-y. Functional source and translation boundary are stated in the revised main text.

Reference 94. Multiple ABCC8 Loss-of-Function Mutations Associated with Paradoxical Maturity-Onset Diabetes of the Young. PMID 42821652. DOI 10.2337/db26-0406. Functional source and translation boundary are stated in the revised main text.

## Prioritised records

PMID 17259403 | 2007 | Type 2 diabetes-associated missense polymorphisms KCNJ11 E23K and ABCC8 A1369S influence progression to diabetes and response to interventions in the Diabetes Prevention Program. | Indexed abstract available

PMID 21989597 | 2012 | Heterozygous ABCC8 mutations are a cause of MODY. | Indexed abstract available

PMID 24814349 | 2015 | Monoallelic ABCC8 mutations are a common cause of diazoxide-unresponsive diffuse form of congenital hyperinsulinism. | Indexed abstract available

PMID 24849284 | 2014 | Carbamazepine inhibits ATP-sensitive potassium channel activity by disrupting channel response to MgADP. | Indexed abstract available

PMID 26682803 | 2015 | The Nucleotide-Binding Sites of SUR1: A Mechanistic Model. | Indexed abstract available

PMID 9287292 | 1997 | MgADP antagonism to Mg2+-independent ATP binding of the sulfonylurea receptor SUR1. | Indexed abstract available

PMID 9135131 | 1997 | The essential role of the Walker A motifs of SUR1 in K-ATP channel activation by Mg-ADP and diazoxide. | Indexed abstract available

PMID 20022885 | 2010 | Interaction between mutations in the slide helix of Kir6.2 associated with neonatal diabetes and neurological symptoms. | Indexed abstract available

PMID 32027066 | 2020 | Update of variants identified in the pancreatic β-cell KATP channel genes KCNJ11 and ABCC8 in individuals with congenital hyperinsulinism and diabetes. | Indexed abstract available

PMID 23275527 | 2013 | Genotype and phenotype correlations in 417 children with congenital hyperinsulinism. | Indexed abstract available

PMID 18708460 | 2008 | DEND mutation in Kir6.2 (KCNJ11) reveals a flexible N-terminal region critical for ATP-sensing of the KATP channel. | Indexed abstract available

PMID 26517901 | 2015 | Molecular action of sulphonylureas on KATP channels: a real partnership between drugs and nucleotides. | Indexed abstract available

PMID 21674179 | 2011 | Hyperinsulinaemic hypoglycaemia and diabetes mellitus due to dominant ABCC8/KCNJ11 mutations. | Indexed abstract available

PMID 20685672 | 2010 | ABCC8 and KCNJ11 molecular spectrum of 109 patients with diazoxide-unresponsive congenital hyperinsulinism. | Indexed abstract available

PMID 17021801 | 2006 | Functional analysis of six Kir6.2 (KCNJ11) mutations causing neonatal diabetes. | Indexed abstract available

PMID 42821652 | 2026 | Multiple ABCC8 Loss-of-Function Mutations Associated with Paradoxical Maturity-Onset Diabetes of the Young. | Indexed abstract available

PMID 28318089 | 2017 | A CACNA1D mutation in a patient with persistent hyperinsulinaemic hypoglycaemia, heart defects, and severe hypotonia. | Indexed abstract available

PMID 29402588 | 2018 | TALK-1 reduces delta-cell endoplasmic reticulum and cytoplasmic calcium levels limiting somatostatin secretion. | Indexed abstract available

PMID 26959991 | 2016 | A Variant of GJD2, Encoding for Connexin 36, Alters the Function of Insulin Producing β-Cells. | Indexed abstract available

PMID 22569071 | 2012 | Impaired β-cell-β-cell coupling mediated by Cx36 gap junctions in prediabetic mice. | Indexed abstract available

PMID 29208420 | 2018 | The inhibitor of connexin Cx36 channels, mefloquine, inhibits voltage-dependent Ca2+ channels and insulin secretion. | Indexed abstract available

PMID 34199454 | 2021 | What Is the Metabolic Amplification of Insulin Secretion and Is It (Still) Relevant? | Indexed abstract available

PMID 19000992 | 2009 | Cx36 makes channels coupling human pancreatic beta-cells, and correlates with insulin expression. | Indexed abstract available

PMID 37578890 | 2023 | Restoring connexin-36 function in diabetogenic environments precludes mouse and human islet dysfunction. | Indexed abstract available

PMID 27806105 | 2016 | Dynamics and Regulation of Insulin Secretion in Pancreatic Islets from Normal Young Children. | Indexed abstract available

PMID 23613279 | 2013 | Hyperglycemia downregulates Connexin36 in pancreatic islets via the upregulation of ICER-1/ICER-1γ. | Indexed abstract available

PMID 28597607 | 2018 | Immaturity of insulin secretion by pancreatic islets isolated from one human neonate. | Indexed abstract available

PMID 22660720 | 2012 | SLC2A2 mutations can cause neonatal diabetes, suggesting GLUT2 may have a role in human insulin secretion. | Indexed abstract available

PMID 22314875 | 2012 | Familial renal glucosuria: a clinicogenetic study of 23 additional cases. | Indexed abstract available

PMID 34171785 | 2021 | An induced pluripotent stem cell line derived from a patient with neonatal diabetes and Fanconi-Bickel syndrome caused by a homozygous mutation in the SLC2A2 gene. | Indexed abstract available

PMID 32913944 | 2020 | Glut1 Deficiency Syndrome (Glut1DS): State of the art in 2020 and recommendations of the international Glut1DS study group. | Indexed abstract available

PMID 28491926 | 2017 | A Novel Intragenic SLC16A1 Mutation Associated With Congenital Hyperinsulinism. | No indexed abstract

PMID 32723473 | 2020 | Cryo-EM structures of human ZnT8 in both outward- and inward-facing conformations. | Indexed abstract available

PMID 10874303 | 2000 | The spectrum of mutations, including four novel ones, in the thiamine-responsive megaloblastic anemia gene SLC19A2 of eight families. | Indexed abstract available

PMID 31676859 | 2019 | Loss of ZnT8 function protects against diabetes by enhanced insulin secretion. | Indexed abstract available

PMID 40853861 | 2026 | Clinical Spectrum of Primary Hypomagnesemia with Secondary Hypocalcemia due to TRPM6 Mutation. | Indexed abstract available

PMID 24172204 | 2014 | H syndrome: the first 79 patients. | Indexed abstract available

PMID 27967291 | 2017 | Novel Hypoglycemia Phenotype in Congenital Hyperinsulinism Due to Dominant Mutations of Uncoupling Protein 2. | Indexed abstract available

PMID 28681398 | 2017 | Analysis of large-scale sequencing cohorts does not support the role of variants in UCP2 as a cause of hyperinsulinaemic hypoglycaemia. | No indexed abstract

PMID 22829870 | 2012 | The mitochondrial Ca2+ uniporter MCU is essential for glucose-induced ATP increases in pancreatic β-cells. | Indexed abstract available

PMID 33932586 | 2021 | Mitochondrial clearance of calcium facilitated by MICU2 controls insulin secretion. | Indexed abstract available

PMID 39069376 | 2024 | PDZD8 Augments Endoplasmic Reticulum-Mitochondria Contact and Regulates Ca2+ Dynamics and Cypd Expression to Induce Pancreatic β-Cell Death during Diabetes. | Indexed abstract available

PMID 42247169 | 2026 | Diabetes-associated MYT1 and ST18 genes regulate human beta cell insulin secretion and survival via other diabetes risk genes. | Indexed abstract available

PMID 28481223 | 2017 | Ca2+ channel clustering with insulin-containing granules is disturbed in type 2 diabetes. | Indexed abstract available

PMID 39313541 | 2024 | Loss of electrical β-cell to δ-cell coupling underlies impaired hypoglycaemia-induced glucagon secretion in type-1 diabetes. | Indexed abstract available

PMID 36347081 | 2022 | The T-type calcium channel CaV3.2 regulates insulin secretion in the pancreatic β-cell. | Indexed abstract available

PMID 40467586 | 2025 | FXYD2 marks and regulates maturity of β cells via ion channel-mediated signal transduction. | Indexed abstract available

PMID 28607108 | 2017 | Kv2.1 Clustering Contributes to Insulin Exocytosis and Rescues Human β-Cell Dysfunction. | Indexed abstract available

PMID 30293774 | 2019 | Preserving Insulin Secretion in Diabetes by Inhibiting VDAC1 Overexpression and Surface Translocation in β Cells. | Indexed abstract available

PMID 33893274 | 2021 | CRISPR-based genome editing in primary human pancreatic islet cells. | Indexed abstract available

PMID 31085831 | 2019 | A glucose-dependent spatial patterning of exocytosis in human β-cells is disrupted in type 2 diabetes. | Indexed abstract available

PMID 29346932 | 2017 | Gap-junction coupling and ATP-sensitive potassium channels in human β-cell clusters: Effects on emergent dynamics. | Indexed abstract available

PMID 32051343 | 2020 | SNAP23 depletion enables more SNAP25/calcium channel excitosome formation to increase insulin exocytosis in type 2 diabetes. | Indexed abstract available

PMID 18243136 | 2008 | Cytosolic adenylate kinases regulate K-ATP channel activity in human beta-cells. | Indexed abstract available

PMID 22492527 | 2012 | Reduced insulin exocytosis in human pancreatic β-cells with gene variants linked to type 2 diabetes. | Indexed abstract available

PMID 26239056 | 2015 | Type 2 Diabetes-Associated K+ Channel TALK-1 Modulates β-Cell Electrical Excitability, Second-Phase Insulin Secretion, and Glucose Homeostasis. | Indexed abstract available

PMID 26862742 | 2016 | Protection of Human Pancreatic Islets from Lipotoxicity by Modulation of the Translocon. | Indexed abstract available

PMID 30412665 | 2019 | Exendin-4 overcomes cytokine-induced decreases in gap junction coupling via protein kinase A and Epac2 in mouse and human islets. | Indexed abstract available

PMID 41606169 | 2026 | Zinc accumulation-induced integrated stress response triggers β-cell identity loss. | Indexed abstract available

PMID 39326417 | 2024 | Transplantation of chemically induced pluripotent stem-cell-derived islets under abdominal anterior rectus sheath in a type 1 diabetes patient. | Indexed abstract available

PMID 40348569 | 2025 | Global Disparities in Congenital Hyperinsulinism Care. | Indexed abstract available

PMID 41201695 | 2026 | Advances in Pharmacotherapy for Congenital Hyperinsulinism. | Indexed abstract available

PMID 29880307 | 2018 | Precision medicine in KCNJ11 permanent neonatal diabetes. | No indexed abstract

PMID 35241836 | 2022 | Functional, metabolic and transcriptional maturation of human pancreatic islets derived from stem cells. | Indexed abstract available

PMID 36537518 | 2022 | ISPAD Clinical Practice Consensus Guidelines 2022: The diagnosis and management of monogenic diabetes in children and adolescents. | No indexed abstract

PMID 40782792 | 2025 | Reconstruction of endocrine subtype-complete human pluripotent stem cell-derived islets with capacity for hypoglycemia protection in vivo. | Indexed abstract available

PMID 28600547 | 2017 | Modeling Congenital Hyperinsulinism with ABCC8-Deficient Human Embryonic Stem Cells Generated by CRISPR/Cas9. | Indexed abstract available

## Author verification

Hajar Dauleh and Khalid Hussain verified the AI-assisted screening, key full-text interpretations and final H1–H5 assignments. This verification was not independent duplicate screening. No formal risk-of-bias assessment was performed for this narrative review.

## Archived retrieval and ordered PMID lists

The lists below are copied directly from each original ESearch response idlist array. Rank is the position in that returned array, beginning at 1; block membership and retrieval order are preserved, including overlap between blocks. Each request used sort=relevance literally. The audit reports this submitted parameter without retrospectively substituting another sort label or claiming independent validation of PubMed ranking. The first-60 cap was applied by retmax=60 in each ESearch request, before cross-block deduplication; retstart was omitted and responses record 0. No additional result pages were fetched.

The publication-date ceiling defines the query range, not comprehensive ascertainment through 23 October 2026. Relevant records, including recent publications, may lie beyond each cap. Ranking and indexing may change; rerunning these queries cannot establish the historical order.

## S1 KATP

1: 17259403; 2: 28123437; 3: 24618446; 4: 34682101; 5: 21989597; 6: 24814349; 7: 28824061; 8: 22796691; 9: 26321954; 10: 31821855.

11: 19500515; 12: 24849284; 13: 26682803; 14: 17919176; 15: 28018462; 16: 34825567; 17: 9287292; 18: 12351459; 19: 9135131; 20: 14645230.

21: 19475716; 22: 9990013; 23: 20022885; 24: 39734857; 25: 35851216; 26: 15916932; 27: 32027066; 28: 23275527; 29: 32104032; 30: 33102403.

31: 15501029; 32: 20550977; 33: 35847046; 34: 29035201; 35: 24421282; 36: 18708460; 37: 22967881; 38: 20938745; 39: 36458573; 40: 23807917.

41: 26517901; 42: 16475928; 43: 11237225; 44: 31137773; 45: 31201216; 46: 21674179; 47: 24621811; 48: 20685672; 49: 19342262; 50: 29594720.

51: 33595839; 52: 34463643; 53: 11425552; 54: 12475777; 55: 17021801; 56: 10805170; 57: 11347455; 58: 39385789; 59: 21422196; 60: 42821652.

## S2 excitable channels

1: 38723859; 2: 22581745; 3: 28649285; 4: 31778749; 5: 22961080; 6: 25369983; 7: 41474140; 8: 35892445; 9: 39457433; 10: 25560470.

11: 34547194; 12: 32273741; 13: 42669336; 14: 20816152; 15: 28607931; 16: 39332965; 17: 31623129; 18: 26679211; 19: 39122224; 20: 28837395.

21: 26911676; 22: 27664711; 23: 15863036; 24: 19247372; 25: 33116037; 26: 19714318; 27: 22308672; 28: 23157812; 29: 32016123; 30: 26803651.

31: 22696034; 32: 41315624; 33: 20570216; 34: 34520625; 35: 35673481; 36: 41287619; 37: 28318089; 38: 30689740; 39: 40313396; 40: 34667723.

41: 24843522; 42: 31515486; 43: 22966882; 44: 22151254; 45: 36929614; 46: 29402588; 47: 19933996; 48: 40502026; 49: 22158537; 50: 27124316.

51: 42014799; 52: 33346478; 53: 39619217; 54: 28246294; 55: 42459212; 56: 23808542; 57: 41539350; 58: 23193183; 59: 23018631; 60: 19443733.

## S3 network

1: 21084853; 2: 27932063; 3: 24096873; 4: 21527868; 5: 41838901; 6: 16931449; 7: 26959991; 8: 22569071; 9: 34763008; 10: 34979329.

11: 29208420; 12: 16263767; 13: 24843702; 14: 40956879; 15: 24326425; 16: 14681053; 17: 33324349; 18: 35987388; 19: 34199454; 20: 18073214.

21: 10905480; 22: 22752717; 23: 34231467; 24: 35114378; 25: 29706896; 26: 12064624; 27: 19817798; 28: 19000992; 29: 38883608; 30: 28006000.

31: 37578890; 32: 27506729; 33: 24069942; 34: 28686518; 35: 32339440; 36: 17395748; 37: 22848521; 38: 23973309; 39: 25788295; 40: 22001400.

41: 27806105; 42: 27851770; 43: 23420660; 44: 30052866; 45: 23831630; 46: 25129107; 47: 24735890; 48: 28081772; 49: 22729650; 50: 35954174.

51: 32688335; 52: 23613279; 53: 42293346; 54: 28284720; 55: 12766175; 56: 15023528; 57: 28597607; 58: 17919186; 59: 22013215; 60: 18258229.

## S4 glucose transport

1: 40053493; 2: 29573118; 3: 33860925; 4: 28657399; 5: 29741207; 6: 37230079; 7: 30616884; 8: 33431108; 9: 22166420; 10: 22660720.

11: 15610225; 12: 30413829; 13: 22831748; 14: 22314875; 15: 23306390; 16: 29207384; 17: 29978611; 18: 17169300; 19: 33659800; 20: 34572917.

21: 21791420; 22: 37542344; 23: 10863940; 24: 34171785; 25: 22802087; 26: 18387950; 27: 28106060; 28: 27825869; 29: 21204808; 30: 30040286.

31: 21546317; 32: 14614622; 33: 12220445; 34: 26215149; 35: 11079206; 36: 36216487; 37: 40405536; 38: 30132033; 39: 38851145; 40: 35323940.

41: 35348743; 42: 35115100; 43: 9815014; 44: 28378819; 45: 39243758; 46: 38834489; 47: 32913944; 48: 38320495; 49: 35077036; 50: 37407157.

51: 40430352; 52: 31208401; 53: 35973390; 54: 36947088; 55: 30559107; 56: 33417139; 57: 26445203; 58: 32748638; 59: 28283348; 60: 35421452.

## S5 metals and carriers

1: 41163347; 2: 29431830; 3: 34547194; 4: 28491926; 5: 18400535; 6: 33666829; 7: 24547910; 8: 30723056; 9: 31951336; 10: 42617928.

11: 32634200; 12: 24072090; 13: 32776596; 14: 32723473; 15: 11286512; 16: 33630370; 17: 18614593; 18: 27363941; 19: 12393806; 20: 18438525.

21: 24675520; 22: 10874303; 23: 28760554; 24: 17293876; 25: 33050689; 26: 31951337; 27: 26316429; 28: 24894595; 29: 34187112; 30: 20140240.

31: 39296124; 32: 37256143; 33: 31676859; 34: 34440664; 35: 23623699; 36: 25793017; 37: 23638917; 38: 26142673; 39: 37324267; 40: 28303020.

41: 25878670; 42: 22576805; 43: 26178371; 44: 31273433; 45: 39619217; 46: 40853861; 47: 31092477; 48: 11380424; 49: 21067978; 50: 30392157.

51: 20424228; 52: 31296181; 53: 27911147; 54: 22526607; 55: 24964825; 56: 38871680; 57: 29280746; 58: 24172204; 59: 22069278; 60: 27112957.

## S6 organelles

1: 26900134; 2: 42503360; 3: 41396300; 4: 28130074; 5: 34547194; 6: 18193644; 7: 20103532; 8: 39153480; 9: 18940651; 10: 28790027.

11: 26316429; 12: 42474271; 13: 17514359; 14: 27967291; 15: 24440978; 16: 22461387; 17: 27796346; 18: 18301352; 19: 26526553; 20: 30917323.

21: 39619217; 22: 15951317; 23: 41300164; 24: 28681398; 25: 22829870; 26: 16679295; 27: 12716765; 28: 35834533; 29: 12401712; 30: 11440717.

31: 16021520; 32: 21172424; 33: 11350740; 34: 22393835; 35: 12743644; 36: 40671041; 37: 16928598; 38: 34698550; 39: 29280746; 40: 32698623.

41: 33499062; 42: 29131158; 43: 33932586; 44: 41102706; 45: 37310861; 46: 29793507; 47: 27076074; 48: 28833790; 49: 33627316; 50: 29493090.

51: 39069376; 52: 16766189; 53: 26323472; 54: 16098826; 55: 19474719; 56: 30100544; 57: 39702527; 58: 25322829; 59: 23132673; 60: 11440712.

## S7 remodelling

1: 42247169; 2: 31676859; 3: 28481223; 4: 29212789; 5: 15000528; 6: 25322829; 7: 36343918; 8: 34978761; 9: 39313541; 10: 30044988.

11: 36347081; 12: 30242153; 13: 40467586; 14: 34979329; 15: 25844899; 16: 38127123; 17: 28975620; 18: 32492936; 19: 28237718; 20: 40108136.

21: 26931137; 22: 28403169; 23: 31871187; 24: 33832651; 25: 33446570; 26: 15483097; 27: 32467389; 28: 28607108; 29: 29180017; 30: 17353295.

31: 30293774; 32: 27533789; 33: 31983511; 34: 32339440; 35: 33893274; 36: 33986325; 37: 37943614; 38: 31085831; 39: 27284112; 40: 29606630.

41: 32432213; 42: 25532126; 43: 29346932; 44: 28768770; 45: 32051343; 46: 18243136; 47: 28951826; 48: 25930156; 49: 26861785; 50: 42150252.

51: 18174159; 52: 28753206; 53: 22492527; 54: 26239056; 55: 21220936; 56: 12196472; 57: 29309562; 58: 30228187; 59: 26862742; 60: 30412665.

## S8 translation

1: 42898925; 2: 38859884; 3: 42871118; 4: 37146579; 5: 14964026; 6: 30881142; 7: 29699867; 8: 20931292; 9: 32699230; 10: 41357370.

11: 41606169; 12: 25238204; 13: 41810512; 14: 8410522; 15: 20878482; 16: 21186002; 17: 42523425; 18: 39153498; 19: 42328330; 20: 35624321.

21: 39441403; 22: 27173951; 23: 35798301; 24: 30114684; 25: 15530324; 26: 39326417; 27: 32595377; 28: 36894549; 29: 35442606; 30: 25905200.

31: 40348569; 32: 39344692; 33: 29406006; 34: 31511734; 35: 40487603; 36: 36170658; 37: 23275527; 38: 35115708; 39: 24865345; 40: 38584412.

41: 38104245; 42: 32333556; 43: 41201695; 44: 36599351; 45: 23345197; 46: 37056678; 47: 29880307; 48: 35241836; 49: 20550977; 50: 21186003.

51: 34469064; 52: 36537518; 53: 32851339; 54: 40782792; 55: 29301189; 56: 31250216; 57: 28600547; 58: 41539914; 59: 33595839; 60: 22315648.

## Audit archive contents and integrity checks

Supplementary Search Audit S2 contains the unmodified 23 October files under original/, including eight block JSON responses, search_log.json, structured_search.py, five PubMed EFetch batches (pubmed_records_0.xml, pubmed_records_100.xml, pubmed_records_200.xml, pubmed_records_300.xml and pubmed_records_400.xml), records.json, journal_record_selection.csv, abstract_selection.json, new_sources.json and three saved PMC full-text XML files. The EFetch batches follow the combined deduplicated retrieval and are not separate block-specific exports. The ordered lists preserve the block membership that the pooled exports alone do not encode.

Checks on 28 October 2026 reconciled all eight 60-ID lists, 480 block-specific records, 467 unique identifiers and 467 XML records. The latter contain 465 journal articles and two book/database records. The prioritisation file contains 67 identifiers, of which 63 have indexed abstracts. Derived files in S2 comprise eight one-PMID-per-line lists, a 480-row block/rank/PMID CSV, this supplement and a SHA-256 manifest. The manifest checks file integrity; it does not establish independent scientific validation. No replacement search was run for this revision.
